# Ligation-Driven Electrochemical Magneto-Genoassay Platform Based on PNA Probes for the Multiple Detection of Soy and Mustard DNA in Wheat Flour

**DOI:** 10.3390/bios16060340

**Published:** 2026-06-16

**Authors:** Simone Fortunati, Shaista Nazir, Federico Biondi, Mattia Amariglio, Eloisa Tosi, Roberto Corradini, Gaetano Donofrio, Francesca Lambertini, Michele Suman, Alex Manicardi, Marco Giannetto, Maria Careri

**Affiliations:** 1 Department of Chemistry, Life Sciences and Environmental Sustainability, University of Parma, Parco Area delle Scienze 17/A, 43124 Parma, Italy; simone.fortunati@unipr.it (S.F.); shaista.nazir@unipr.it (S.N.); federico.biondi@unipr.it (F.B.); mattia.amariglio@unipr.it (M.A.); eloisa.tosi@unipr.it (E.T.); roberto.corradini@unipr.it (R.C.); alex.manicardi@unipr.it (A.M.); maria.careri@unipr.it (M.C.); 2 Biostructures and Biosystems National Institute (I.N.B.B. Consortium), Viale delle Medaglie d’Oro 305, 00136 Roma, Italy; 3 Department of Veterinary Science, University of Parma, Strada del Taglio 10, 43126 Parma, Italy; gaetano.donofrio@unipr.it; 4 Barilla G.R. F.lli SpA, Research, Development & Quality, Sensory and Analytical Food Science, Via Mantova 166, 43122 Parma, Italy; francesca.lambertini@barilla.com (F.L.); michele.suman@barilla.com (M.S.); 5 Department of Food and Drug, University of Parma, Parco Area delle Scienze 27/A, 43124 Parma, Italy

**Keywords:** food allergens, electrochemical sensors, DNA detection, PNA probes, bio-orthogonal ligation, soy, mustard

## Abstract

Food allergies are one of the most critical food safety issues, with epidemiological studies confirming a global increase. In this context, effective and sensitive analytical methods play a crucial role in ensuring allergen-free food products. To face this issue, electrochemical biosensors offer powerful, sensitive, selective, and cost-effective alternatives to conventional methods for food allergen analysis while enabling rapid on-site detection. In this study, we developed a sandwich electrochemical magneto-genoassay aimed at the parallel detection of soy (*Glycine max*) and mustard (*Sinapis alba*) allergens, suitable for implementation on multichannel instrumentation. The assay involves the functionalization of magnetic microbeads functionalized with peptide nucleic acid-based (PNA) capture probes, capable of undergoing target-induced bio-orthogonal ligation with biotin-labelled signalling probes. Carbon nanotubes-modified screen-printed carbon electrodes were exploited for the voltammetric readout. We demonstrated the effectiveness of functional PNA probes by comparing their performance with those achieved using analogous DNA probes. The developed method exhibited excellent selectivity in terms of cross-reactivity, sensitivity, and precision, achieving detection limits of 16 and 19 pM for soy and mustard, respectively. Finally, by successfully applying the biosensor platform to genomic DNA extracted from plant-based food ingredients, we demonstrated its potential as a valuable tool in food safety risk management.

## 1. Introduction

Food allergens are among the most critical hazards in food safety monitoring, as even trace amounts can trigger severe and potentially life-threatening reactions in sensitized individuals. Epidemiological studies confirm a global increase in food allergies, driven by a complex interaction of genetic, epigenetic, and environmental factors [1].

Despite advances in treatment, including immunotherapy for peanut allergy and recently FDA-approved drugs that target multiple allergens [2], management still relies on strict allergen avoidance and emergency epinephrine administration, with a significant impact on patients and healthcare systems.

According to Regulation (EU) No. 1169/2011 (as amended by Commission Delegated Regulation No. 78/2014), 14 allergenic ingredients must be declared on labels, including the “Big 8” (gluten cereals, crustaceans, eggs, fish, peanuts, soybeans, milk, tree nuts) and additional allergens such as celery, mustard, sesame, lupin, mollusks, and sulphur dioxide/sulphites. However, the widespread voluntary use of precautionary statements (e.g., “may contain”) raises concerns due to the potential for consumer misunderstanding and understatement of risk [3]. The issue is further complicated by accidental exposure from undeclared ingredients, adulteration, emerging allergens, and cross-contamination during production and processing [4,5].

In this scenario, reliable analytical methods for allergen detection play a crucial role in ensuring safety of food products. Current approaches, including immunoassays, DNA-based assays, and mass spectrometry (MS), target markers of the presence of allergenic ingredients, i.e., proteins, DNA, or peptides, with high sensitivity requirements [5,6,7,8]. Among protein-targeting approaches, including MS-based techniques and Enzyme-Linked Immunosorbent Assays (ELISA) tests, liquid chromatography (LC)-MS/MS emerges as the gold standard due to its high sensitivity and selectivity even for trace amounts of allergens in complex food matrices. This technique enables unambiguous protein identification and the accurate quantification of proteins [7,8,9], although its application is limited by cost and the expertise required. ELISA methods are widely used for routine monitoring due to their high throughput and simple analytical workflow, but suffer from limitations such as cross-reactivity, matrix effects, and reduced reliability for processed or thermally labile proteins [10]. Even though LC-MS-based methods are well-established for superior multiplexing capability in allergen analysis compared to ELISA, both methods may suffer limitations for food allergen quantitation, specifically with respect to sample extraction [8,9,10,11,12].

To address protein degradation issues in processed food samples, analytical approaches relying on DNA recognition provide a promising alternative [6]. In this context, real-time polymerase chain reaction (PCR) offers high sensitivity and specificity for allergen detection [5,7], though it requires optimized DNA extraction and carefully designed primers and probes. In addition, the lack of portability prevents its implementation throughout the food chain [13], although recent microfluidic platforms have enabled integrated, on-site multiplex detection within two hours [14].

In this context, biosensing approaches represent a promising alternative, offering high sensitivity, selectivity, low cost, and rapid on-site detection [15,16]. These systems combine transducers with bio-recognition elements and benefit from advanced nanomaterials that enhance signal and electron transfer [15,16,17].

In particular, electrochemical nucleic acid-based sensors (genosensors) enable highly sensitive and selective allergen DNA detection with rapid response and potential for point-of-need testing [18,19,20]. Compared to immunosensors, they provide improved specificity and robustness, as they are less prone to matrix effects [21]. They also support portable, low-cost, and automated platforms, compatible with IoT integration [22], and enable PCR-free detection with minimal sample preparation [18,23,24]. Their performance can be further improved by adoption of magnetic supports onto which the bioassay can be performed, thus allowing a fine control of the working conditions and avoiding modification of the transducer’s surface [25,26,27,28]. Furthermore, their multiplexing capability allows the simultaneous detection of multiple allergens, improving throughput and reducing costs [18,29]. While conventional genosensors rely on DNA probes, improved analytical performance can be achieved by implementing peptide nucleic acids (PNAs). Indeed, PNAs are characterized by a neutral pseudopeptide backbone and bind complementary DNA with greater stability and specificity, allowing for shorter probes and better mismatch discrimination [30]. PNA probes can be easily synthesized via solid-phase methods [31] and are increasingly used in detection platforms, including microarray-based allergen analysis [32,33]. A common approach in electrochemical genosensing is based on the formation of a sandwich complex to achieve target DNA capture and output signal generation [18,34,35]. This architecture is based on the simultaneous hybridization of the target DNA in two different adjacent regions by a capture probe (CP), responsible for immobilizing the complex on a solid support, and a signalling probe (SP), which is modified with a tag that enables signal generation. However, approaches based solely on reversible probes and target hybridization can suffer from signal loss associated with repeated washing steps and variations in temperature and solution variations. In contrast, ligation between probes converts a reversible recognition event into an irreversible covalent bond, allowing the use of stringent assay conditions without compromising the readout sensitivity, as the signal does not depend only on a hybridization equilibrium relying on non-covalent interactions. We recently reported bio-orthogonal ligation chemistry between a 1,4-diketone, namely 2,5-dioxopentanyl (DOP) and α-nucleophiles (α-Nu) that proceeds only in the presence of proximity, with high specificity, yielding a stable and robust ligation product [36,37,38]. In the context of sandwich hybridization assays, the implementation of probes capable of bio-orthogonal ligation holds great promise for improving analytical performance. In fact, the covalent linking of the probes, which is triggered by target recognition, prevents potential signal loss in the subsequent steps that are commonly involved in sandwich assays, such as enzymatic labelling.

To the best of our knowledge, previous studies focused on amplification-free electrochemical genosensors for allergen detection have involved both label-free approaches with impedimetric readout and enzyme-labelled approaches, both based on stem-loop probes. Sun et al. detected DNA sequences associated with peanut allergen using complex architectures and electrode functionalization with advanced composite nanomaterials [39,40,41]. Although these approaches achieve very low detection limits, the use of multiple nanomaterials for probe immobilization and signal amplification makes them time-consuming and costly. Furthermore, the use of a single stem-loop probe results in a less specific response than a sandwich approach involving two recognition events. In this context, Gamella et al. developed an innovative approach based on a DNA/RNA heteroduplex sandwich for targeting the mustard Sin a1 sequence [24]. Two different antibodies were used to generate the electrochemical signal: the first antibody recognizes the heteroduplex, and the second is a secondary antibody labelled with the peroxidase enzyme. While the method achieves acceptable detection limits, it has significant drawbacks, including high methodological complexity, the high cost of antibodies, and the lability of the RNA probe.

In this study, we developed, for the first time, a genosensing platform for multi-allergen detection based on a sandwich electrochemical PNA ligation magneto-genoassay protocol tailored for multichannel instrumentation. The assay involves the immobilization of PNA capture probes, modified with a moiety capable of undergoing target-induced bio-orthogonal ligation with biotin-labelled signalling probes, on magnetic microbeads (MBs). After carefully selecting DNA sequences attributable to soy and mustard, we applied the method to simultaneously detect the two allergens. To this end, a voltammetric readout implemented on carbon nanotubes-modified screen-printed carbon electrodes was performed. A streptavidin–alkaline phosphatase conjugate was used to enable the processing of hydroquinone diphosphate as an enzyme substrate, thus generating electroactive hydroquinone in the presence of the target DNA. The effectiveness of PNA probes and bio-orthogonal ligation was demonstrated by comparing the performance of the genoassay with that achieved using (longer) DNA probes with analogous sequences or unreactive PNA probes.

The implementation of ligation-capable PNA probes for the selective recognition of genomic DNA extracted from plant-based food ingredients, combined with a systematic evaluation of restriction enzymes for sequence-dependent genomic DNA digestion, represents an unprecedented methodological approach. A further advantage of the proposed approach lies in its high versatility, enabling its integration into multichannel electroanalytical platforms for the simultaneous detection of multiple target allergens. Therefore, both the development of PNA probes capable of target-induced ligation and the design of a multichannel electrochemical magneto-genoassay for multi-allergen detection constitute distinctive and innovative aspects of the present work, which significantly advance the current state of the art in electrochemical genoassays for food allergen analysis.

## 2. Materials and Methods

### 2.1. Materials and Equipment

Sodium chloride (NaCl), disodium hydrogen phosphate (Na_2_HPO_4_), ethylendiaminetetraacetic acid disodium salt dihydrate (Na_2_EDTA), sodium bicarbonate (NaHCO_3_), Trizma^®^ Base, magnesium chloride (MgCl_2_), N-(3-dimethylaminopropyl)-N′-ethylcarbodiimide hydrochloride (EDC), N-hydroxysuccinimide (NHS), 4- morpholineethanesulfonic acid monohydrate (MES), Tween^®^ 20, bovine serum albumin (BSA), hydrochloric acid (HCl, 37%), sodium hydroxide (NaOH), and streptavidin–alkaline phosphatase from *Streptomyces avidinii* conjugate (ALP-Strp) and the endonuclease enzymes HindIII, BamHI, MnlI, DpnI, MseI, TruI, and DNAse I were purchased from Sigma-Aldrich (Milan, Italy).

Soy (*Glycine max*, GM), mustard (*Sinapis alba*, SA), and wheat (*Triticum turgidum durum*, TTD) samples were supplied by Barilla G.R. F.lli SpA (Parma, Italy). Synthetic GM and SA DNAs were purchased from Metabion GmbH (Planegg, Germany). “IONspin K DNA Prep One-for-All” was purchased from GENERON (Modena, Italy).

Micro-magnetic beads Dynabeads™ M-270 carboxylic acid were purchased from Thermo Fisher Scientific (Waltham, MA, USA). Hydroquinone diphosphate (HQDP), single-walled carbon nanotubes-modified screen-printed carbon electrodes DRP-110SWCNT (SWCNT-SPEs) with 0.2 cm radius working electrode and silver pseudoreference electrode, magnetic support for screen-printed electrodes (DRP-magnet), and support for magnetic separation were supplied by Metrohm Italiana Srl (Origgio, Varese, Italy). ThermoMixer^®^ C was purchased from Eppendorf (Milan, Italy). Spark^®^ Multimode Microplate Reader and NanoQuant Plate™ were purchased from Tecan (Männedorf, Switzerland). The voltammetric readout was performed using a μStat 8000 MultiPotentiostat/Galvanostat (Metrohm Italiana Srl, Origgio, Varese, Italy) using DropView 8400M Software. The SPEs were connected to a switch box from DropSens (DRP-DSC), allowing their interface with the potentiostat.

### 2.2. Buffer Solutions’ Composition

Buffer solutions were prepared according to the following compositions:

- MES buffer: 0.025 M MES (pH adjusted to 5.0 using NaOH).
- Activation buffer: 25 mg/mL (0.13 M) EDC and 25 mg/mL (0.22 M) NHS in MES buffer.
- Tris-buffered saline (TBS): 0.1 M Trizma^®^ base, 0.02 M MgCl_2_ (pH adjusted to 7.4 using HCl). Tris-buffered saline–Tween (TBS-T): 0.1 M Trizma^®^ base, 0.02 M MgCl_2_, 0.05% *w*/*v* Tween 20^®^ (pH adjusted to 7.4 using HCl).
- Hybridization buffer (HB): 0.3 M NaCl, 0.02 M Na_2_HPO_4_, 0.1 mM EDTA (pH adjusted to 7.4 using HCl).
- Blocking buffer (BB): 30 mg/mL BSA in TBS (pH 7.4).
- Enzyme buffer (EB): 20 mg/mL BSA in TBS (pH 7.4).
- Reading buffer (RB): 0.1 M Trizma^®^ base, 0.02 M MgCl_2_ (pH adjusted to 9.8 using HCl).

### 2.3. PNA Synthesis

Full PNA synthesis protocols are reported in the Supporting Information. Briefly, PNAs were synthesized using a Rink Amide ProTide resin preloaded with Fmoc-Lys(Mtt)-OH as first monomer (0.2 mmol/g). PNAs were synthesized following an automated Fmoc-based synthesis protocol [42]. Modifications were introduced manually after removal of Fmoc (20% piperidine, 2 × 8 min) or Mtt (HFIP/DCM 1:1, containing 90 mM HOBt, 4 × 3 min) protective groups, allowing selective introduction of the functional groups at the N- or C-terminus, respectively. 3-(5-methylfuran-2-yl)propanoic acid was used as a building block for the introduction of the DOP moiety [36]. PNAs were cleaved from the solid support using a TFA/m-cresol/thioanisole (8:1:1, *v*/*v*/*v*) cleavage cocktail and then precipitated in diethyl ether. Probes were then dissolved in water and purified by reversed-phase high-performance liquid chromatography (RP-HPLC). PNA purity and identity were assessed by HPLC–UV–mass spectrometry (MS); yield was determined after solubilization in milliQ water and calculating PNA concentration from the absorbance at 260 nm using Lambert–Beer’s law (see Supporting Information).

### 2.4. Magneto-Genoassay Development

The magneto-genoassay was performed following the general protocol described below.

#### 2.4.1. Capture Probe Immobilization on MBs

An aqueous suspension of 8 × 10^7^ beads/mL Dynabeads™ M-270 carboxylic acid magnetic beads was obtained by dilution in 100 μL MES buffer. The suspension was then treated according to the washing protocol, which consisted of resuspension in 200 μL of MES buffer followed by magnetic separation for 2 min and removal of the supernatant. Activation of the carboxyl functionalities was performed in accordance with the manufacturer’s procedure by resuspending the beads in 200 μL of activation buffer for 30 min under shaking at 1500 rpm at 25 °C (Thermomixer C). Following a wash with MES buffer, the beads were resuspended in a 200 nM solution of the selected CP sequence in MES buffer, and the reaction was performed over 2 h at 25 °C under shaking at 1500 rpm. The beads modified with the CP were then washed with MES buffer, and their surface was blocked by resuspending them in 200 μL of blocking buffer for 30 min at 25 °C and 1500 rpm. Finally, the beads were washed with TBS and stored at +4 °C.

#### 2.4.2. Sandwich Complex Formation

The DNA samples, either synthetic or obtained from genomic DNA extraction, were mixed at the desired concentration with 200 nM SP in HB. Subsequently, the CP-modified MBs were resuspended in 200 μL of the DNA and SP solution mixture for 1 h at 45 °C while shaking at 1500 rpm. The beads were then washed twice with TBS.

#### 2.4.3. Enzymatic Labelling and Electrochemical Response

The beads were resuspended in 200 μL of a 1:100 ALP-Strp solution diluted in EB for 15 min at 37 °C while shaking at 1500 rpm, after which, they were washed first with TBS-t and then twice with TBS. The beads were then resuspended in 100 μL of a 1 mg/mL solution of HQDP in RB for 5 min at 37 °C, then 50 μL of the suspension were drop-casted onto the SWCNT-SPE placed on the magnetic support for SPE to confine the magnetic particles to the working electrode surface. After preconditioning the electrochemical cell by applying a potential of −0.5 V for 30 s, DPV measurements were performed in the −0.5–+0.2 V potential range using the following parameters: step potential: 0.005 V; modulation amplitude: 0.05 V; modulation time: 100 ms; interval time: 400 ms.

### 2.5. Genomic DNA Treatment

Genomic DNA was extracted from soy granola (*Glycine max*), mustard seeds (*Sinapis alba*), and wheat flour (*Triticum turgidum durum*). Seeds or granola were homogenized in a potter with liquid nitrogen to avoid thermal degradation. The DNA extraction was carried out according to IONspin K DNA Prep One-for-All protocol. The concentration of all genomic DNA extracts was assessed spectrophotometrically by measuring the absorbance at 260 nm using a Spark^®^ Multimode Microplate Reader equipped with the NanoQuant Plate™ to perform micro-volume measurements. DNA concentration was calculated using the Lambert–Beer law, applying the conversion factor of 50 ng/µL per optical density unit at 260 nm for double-stranded DNA [43].

Prior to analysis, genomic DNA (up to 30 µg) was digested for 2 h using 20 units of one of the following restriction enzymes: DpnI, MnlI, TasI, HindIII, BamHI, or MseI. These were selected because they do not cut the target sequence (as predicted by Webcutter 2.0 [44]) but only cleave the surrounding genomic DNA, releasing it, and thus improving access to the target region. As an alternative approach, the same amount of DNA was partially digested using 3 units of DNase I for 3 h. All enzymatic reactions were performed according to the manufacturer’s instructions (Thermo Fisher Scientific, Milan, Italy).

Subsequently, to thermally denature the double-stranded DNA, the mixture was heated to 95 °C for 15 min followed by rapid cooling in an ice bath for 5 min. Strand annealing was retarded by keeping the samples on ice until further steps.

### 2.6. Genoassay Validation

Validation of the developed genoassay was performed on synthetic target DNA for each allergen. Namely, limits of detection (LOD), limits of quantitation (LOQ), precision, and trueness were assessed according to Eurachem Guidelines [45]. For the determination of LOD and LOQ, 10 replicate measurements of blank samples containing no detectable target were acquired. LOD was calculated as 3·*s*_0_/*√n* and LOQ as 10·*s*_0_/*√n*, where *s*_0_ is the blank standard deviation and *n* is the number of replicate measurements. Linearity was calculated by performing three replicated measurements for each concentration level explored in the 50–2000 pM range for GM and in the 100–1500 pM range for SA. The assessment of linearity was based on the calculation of regression residuals, the mean of which was found to not be significantly different from zero (*p* > 0.05) over the linearity range explored.

Precision was assessed in terms of intra-batch and inter-batch repeatability from three replicated measurements for each concentration level within the linear response range. For intra-batch repeatability, replicated measurements were obtained from a single magneto-genoassay, while for inter-batch repeatability, measurements were performed on independent magneto-genoassays on different days.

Trueness was assessed in terms of recovery rates by performing three replicated measurements of two concentration levels of synthetic target DNA for each allergen.

Selectivity was evaluated by performing the magneto-genoassay against 1 nM concentration of non-complementary DNA and comparing the results with those obtained in the absence of the target DNA (negative).

## 3. Results

### 3.1. Electrochemical Magneto-Genoassay Setup and Probes Design

**Scheme 1:**
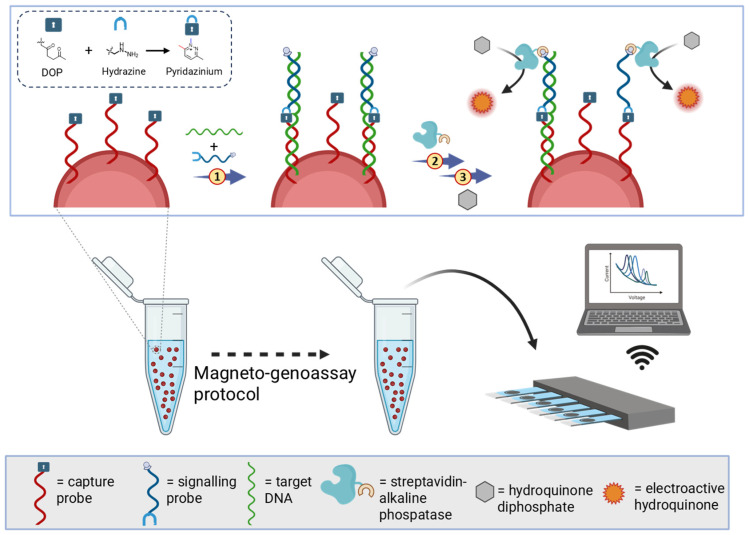
Schematic representation of the electrochemical magneto-genoassay. (1) Simultaneous incubation of target DNA and functional PNA SP on MB modified with functional PNA CP. (2) Enzymatic labelling of biotin-containing SP with streptavidin–alkaline phosphatase enzyme conjugate. (3) Enzymatic dephosphorylation of hydroquinone diphosphate to hydroquinone.

For the development of the electrochemical magneto-genoassay, a sandwich protocol was adopted (Scheme 1) [34,35]. Briefly, the target sequence is simultaneously hybridized in two different regions by a capture probe, covalently bound to the surface of a MB and a biotin-tagged signalling probe. This CP/target/SP sandwich complex is subsequently labelled using a streptavidin–alkaline phosphatase conjugate which enables enzymatic processing of the hydroquinone diphosphate substrate, thus generating electroactive hydroquinone. Finally, a voltammetric readout is performed on screen-printed carbon electrodes modified with single-walled carbon nanotubes. The selection of carbon nanotubes as nanomaterials for the screen-printed electrodes used in the magneto-genoassay readout significantly enhances its sensitivity. In fact, even though the allergen DNA recognition process occurs entirely on the CP-functionalised magnetic microbeads via target-induced ligation between the CP and SP, single-walled carbon nanotubes still provide an intrinsic amplification effect on the electron transfer process, and consequently on the analytical signal, at the same concentration of the enzymatically generated hydroquinone. This behaviour is consistent with our previous studies [34], in which different electrode materials were comparatively evaluated, highlighting the superior electrochemical transduction capability of carbon nanotubes-modified platforms.

The target regions of the probes for soybean (*Glycine max*) were selected in the Lectin 1 gene. For yellow mustard (*Sinapis alba*), the target region was selected in the region of the allergenic storage protein Sin a I gene which has the greatest dissimilarity with Napin storage proteins genes of rapeseed (*Brassica napus*, see Supporting Information Figure S6).

DNA probes were designed to ensure a five-nucleotide gap between the target region of the capture probe and the target region of the signalling probe. In particular, soy-specific DNA probes were obtained from sequences already targeted by previous studies reported in the literature [46]. In contrast, PNA probes were designed in contiguous regions to ensure the proximity of the reactive functions required for ligation. Both 11mer and 15mer PNA sequences were designed to maintain the reactive functions in the same position with respect to the target DNA sequence. In all cases, a Basic Local Alignment Search Tool (BLAST) analysis was performed to exclude potential interference from the wheat (*Triticum aestivum*, *Triticum turgidum durum*) genome. For PNA probes, both parallel and antiparallel orientations were evaluated, as well as possible partial complementarity in both orientations.

PNA capture probes were designed to display a free amino function on a lysine side chain positioned at the C-terminus to allow covalent immobilization on carboxy-functionalized microbeads via amide coupling. Two AEEA (2-[2-[2-aminoethoxy]ethoxy]acetic acid) spacer units were inserted to increase the distance between the PNA sequence and the microbead surface, thereby reducing steric hindrance and enhancing hybridization efficiency [47,48]. The DOP moiety forming the ligation was placed at the N-terminus of the capture probe. In turn, PNA signalling probes were functionalized at their N-terminus with a biotin unit, outdistanced from the PNA sequence with two AEEA spacers to facilitate interaction with streptavidin. The required hydrazine function on a lysine side chain was placed at the C-terminus. This design ensures that, upon recognition of the target DNA, the hydrazine function present on the reported probes is brought into close proximity to the DOP function of the capture probe and triggers the formation of a stable pyridazium bond, which covalently link the biotin moiety to the magnetic beads. To evaluate the efficiency of the bio-orthogonal ligation, control probes bearing acetylated ends were used in place of the reactive hydrazine or DOP functionalities.

The target sequences and the probes used in the study are reported in Table 1.

### 3.2. Working Conditions of the Electrochemical Magneto-Genoassays

After selecting the specific target sequences for the two allergens and designing the probe sequences accordingly, we first verified the effectiveness of the sandwich assay in recognizing the 45mer *Glycine max* target DNA sequence using the 20mer GM-CP-DNA immobilized on the surface of magnetic MBs and 20mer GM-SP-DNA. Similar experiments were carried out for the synthetic *Sinapis alba* DNA-SA sequence using SA-CP-DNA and SA-SP-DNA probes.

Based on previous findings [34], hybridisation experiments of synthetic DNA sequence were performed, varying the concentrations of both CP and SP DNAs in the 100–500 nM range. For each experiment, the target DNA concentration was set at 500 nM (positive) and compared to the signals obtained in the absence of target DNA (negative) to identify the experimental conditions that maximize the positive-to-negative ratio (P/N ratio). The results, shown in Figure S1, confirmed the effectiveness of the sandwich format of the genoassay, with an optimal P/N ratio for DNA CP and DNA SP concentrations of 200 nM.

**Figure 1:**
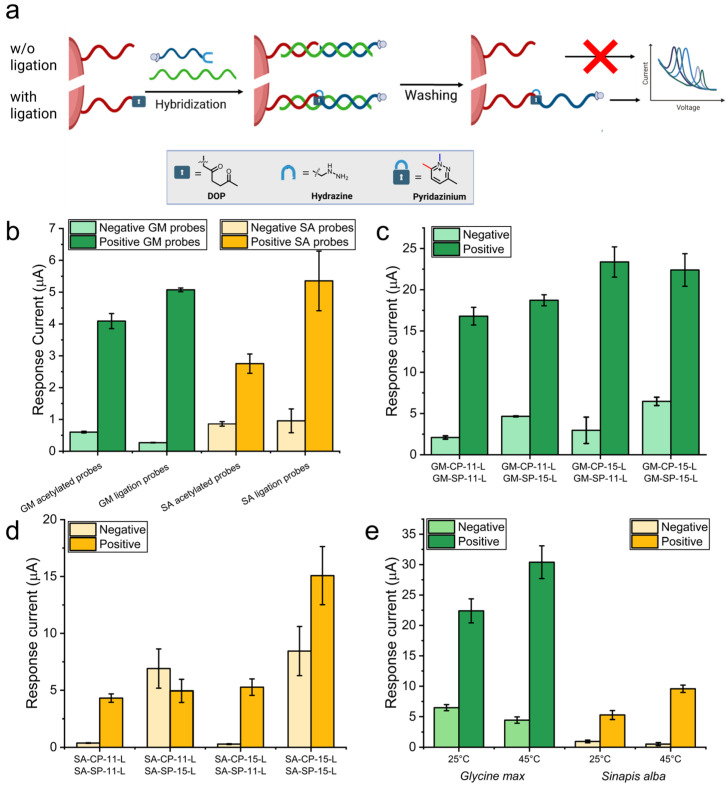
Study of the magneto-genoassays experimental conditions. For all plots, mean current values and standard deviation from three replicated measurements are shown. (**a**) Schematic representation of the magneto-genoassay performed with unmodified PNA probes and with bio-orthogonal ligation-capable probes. (**b**) Effect of ligation on assay performance assessed by comparing the results obtained in the presence (ligation probes) and absence (acetylated probes) of the ligation functionalities. Data are reported for probes intended for the detection of *Sinapis alba* (SA) and *Glycine max* (GM) in the presence (positive) and absence (negative) of target DNA. (**c**) Effect of the length of CP and SP probes on the detection of GM target DNA and their negative controls. (**d**) Effect of the length of CP and SP on the detection of SA target DNA and their negative controls. (**e**) Effect of the increase in hybridization temperature on the magneto-genoassay response to GM and SA target DNA.

To evaluate the performance of the PNA-based probes, responses obtained in the presence and absence of target DNA were acquired using the pair GM-CP-11-L/GM-SP-11-L and SA-CP-11-L/SA-SP-11-L for soy and mustard, respectively. To evaluate the effect of the ligation reaction on the magneto-genoassay performance (Figure 1a), measurements were also performed using PNA probes lacking the required reactive functions (pair GM-CP-11-Ac/GM-SP-11-Ac and SA-CP-11-Ac/SA-SP-11-Ac for soy and mustard, respectively). Based on the previous results obtained with DNA probes, magneto-genoassays were performed by setting the concentration of PNA probes to 200 nM.

The data reported in Figure 1b show P/N ratios of 18 and 6 for the ligation-capable probes targeting GM-T-DNA and SA-T-DNA, respectively, which were significantly higher than the values obtained with the acetylated PNA probes (P/N of 6 and 3, respectively). The improvement observed for reactive probes compared to the standard PNA probes, which are well-known for their superior hybridization properties, was attributed to the target-induced covalent ligation between the hydrazine-modified signalling probes and the DOP-bearing capture probes. Indeed, the ligation reaction ensures signal retention even in the case of wash-induced target DNA melting, resulting in dissociation of the formed sandwich complex (Scheme 1).

It is worth pointing out that, although the hybridisation enhancement effect obtained with PNA probes is apparent in terms of the P/N ratio, the absolute current values relative to positive signals are lower than those obtained with the magneto-genoassay based on DNA probes. In an effort to improve target binding efficiency, longer PNA probes (15mer) were designed for both *Glycine max* (GM-CP-15-L and GM-SP-15-L) and *Sinapis alba* (SA-CP-15-L and SA-SP-15-L) targets, and the effect of combining the two series of probes was evaluated using 5 nM target DNAs.

As shown in Figure 1c, for the *Glycine max* magneto-genoassay, switching from GM-CP-11-L and GM-SP-11-L to a combination of GM-CP-15-L and GM-SP-11-L resulted in a 30% increase in positive signal with no significant change (*p* > 0.05) in the negative control signal. The use of both GM-CP-15-L and GM-SP-15-L led to an increase in negative signal without an improvement in positive response. Interestingly, the elongation of the SP had a modest impact on assay sensitivity, as the use of GM-SP-15-L resulted in only a 10% increase in positive signal compared to the shorter GM-SP-11-L. These data suggest that recognition between the target DNA and CP, which occurs at the interface between the solution and the surface of the magnetic microbeads, benefits from longer probes with greater duplex stability. On the other hand, recognition between the target DNA and SP already reaches maximum efficiency with an 11mer probe, and an elongation of the SP results only in additional non-specific interactions, increasing the negative signal. This behaviour is confirmed in the experiment performed with GM-CP-11-L and GM-SP-15-L, where the short CP limits the positive signal while a two-fold increase in the negative signal is observed as a consequence of the elongation of the SP.

Similar results were obtained on *Sinapis alba* DNA, where the best performance was obtained using SA-CP-15-L and SA-SP-11-L. In fact, their combination produced a moderate increase (≈23%) in the positive signal compared to the results obtained with SA-CP-11-L and SA-SP-11-L. Conversely, using SA-SP-15-L in combination with both CPs significantly increased the negative control signals. These results, shown in Figure 1d, can be ascribed to a partial complementarity between SA-SP-15-L and the CPs.

It should be noted that lower output signals were obtained for *Sinapis alba* than for *Glycine max*. In an effort to improve the magneto-genoassay performance, the effect of hybridisation temperature on the electrochemical signal was evaluated for the detection of both targets (Figure 1e). In both cases, increasing the hybridization temperature from 25 °C to 45 °C resulted in a dual effect: (i) a reduction in the negative signal and (ii) an increase in the positive response. In particular, for the *Sinapis alba* target, a two-fold increase in the positive signal was obtained. Similarly, for the magneto-genoassay based on GM-CP-15-L and GM-SP-11-L, a 25% increase in the positive response was observed at 45 °C. These improvements were ascribed to an enhancement of the hybridisation kinetics, favoured by the superior stability of the PNA-DNA duplexes, in combination with the reduction in non-specific interaction.

### 3.3. Analytical Performance of Magneto-Genoassay for the Detection of Glycine max and Sinapis alba

The magneto-genoassays developed using ligation-capable PNA probes were tested to assess their analytical performance for the measurement of the relevant synthetic target DNA. Results in terms of LOD, LOQ, and linear range are given in Table 2.

Regarding the *Glycine max* target, LOD and LOQ values at 16 and 48 pM were calculated. Accordingly, a GM-T-DNA concentration range from 50 pM to 2 nM was explored, observing a linear response range between 50 pM and 1 nM (Figure S2a). Good intra-batch repeatability was observed, with RSD values lower than 10%. As for inter-batch precision, RSD% values were found to be lower than 15%. The assay demonstrated good trueness, with recovery values from GM-T-DNA at known concentrations (200 and 600 pM) of 95(±4)% and 109(±3)%.

Comparable analytical performance results for the magneto-genoassay aimed at detecting the *Sinapis alba* sequence (Figure S2b) were obtained, with LOD and LOQ values of 19 and 59 pM, respectively. Exploring the concentration of SA-T-DNA in the 100 pM to 1.5 nM range, linearity between 100 pM and 1 nM was observed. Even better precision was obtained, with RSD% values lower than 8% and 10% for intra-batch and inter-batch measurements, respectively. Recovery values of 101(±4)% and 104(±6)% were calculated for target DNA concentrations of 300 pM and 800 pM, respectively, attesting good trueness for SA measurements also.

Furthermore, as shown in Table 2, the use of PNA probes in the magneto-genoassays developed demonstrated superior performance compared to DNA probes for the detection of both *Glycine max* (Figure S3a) and *Sinapis alba* (Figure S3b) targets.

Finally, to demonstrate the selectivity of the assay devised using ligation-capable PNA probes, evaluation of cross-reactivity was carried out by testing the response of GM-CP-15-L and GM-SP-11-L to the non-complementary Sinapis alba target DNA and the response of SA-CP-15-L and SA-SP-11-L to the non-complementary Glycine max target DNA. In both cross-reactivity tests, signals not significantly different (*p* > 0.05) from the negative controls (Figure S4) were observed, thus confirming the specificity of the response towards the intended allergen even in samples containing multiple allergens.

Compared to electrochemical genosensors previously reported in the literature for allergen DNA detection, the proposed magneto-genoassay demonstrates significant advancements over the current state of the art (Table 3). Indeed, many approaches still rely on the adoption of a PCR-based amplification step prior to analysis [49,50,51,52], which has a negative impact on the time, complexity, and cost of each assay. Amplification-free strategies have also been developed, including the magneto-genoassay aimed at mustard detection reported by Gamella and coworkers [24], which drawbacks have been discussed in the Introduction. It should be noted that, while the magneto-genoassay developed in this study allows allergen DNA detection in just over an hour, some approaches reported in the literature achieved faster analysis times. Nevertheless, considering the overall performance, the proposed magneto-genoassay emerges as a valuable analytical technique.

### 3.4. Development of PNA-Based Magneto-Genoassay for the Analysis of Genomic DNA Extracted from Plant-Based Food Ingredients

**Figure 2:**
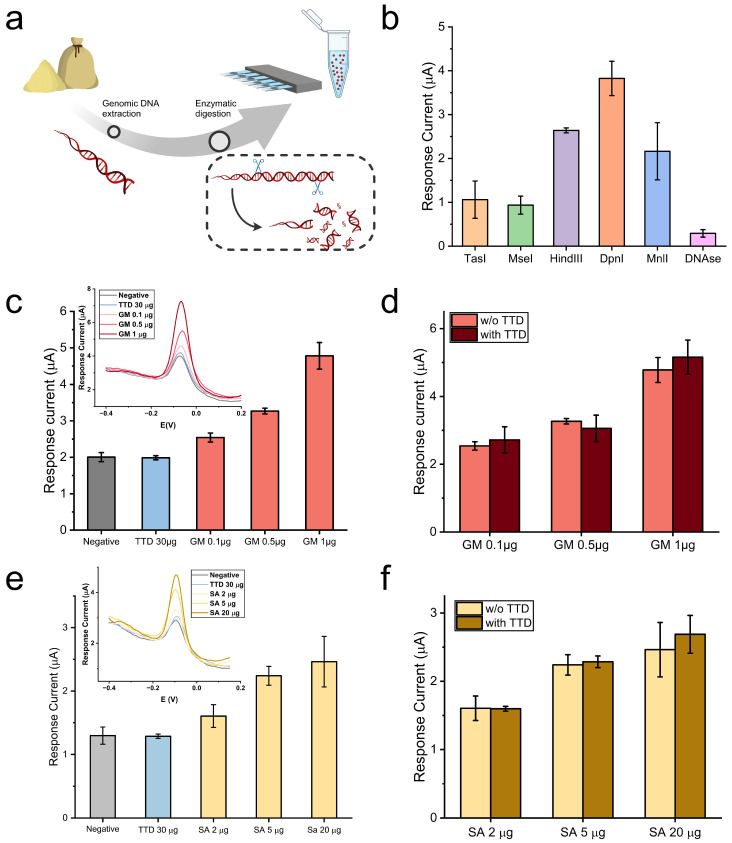
(**a**) Schematic representation of the magneto-genoassay workflow for genomic DNA detection. (**b**) Comparison of the results obtained by analyzing *Glycine max* genomic DNA digested with different endonuclease enzymes. (**c**) Results obtained by testing digested genomic DNAs of TTD (blue bar) and different quantities of GM (red bars) using the magneto-genoassay for GM detection. Inset: relevant voltammograms acquired in each of the conditions reported. (**d**) Results obtained analyzing different quantities of GM genomic DNA, pure and mixed, with 30 µg of *Triticum turgidum durum* genomic DNA. (**e**) Results obtained by testing digested genomic DNAs of TTD (blue bar) and different quantities of SA (yellow bars) using the magneto-genoassay for SA detection. Inset: relevant voltammograms acquired in each of the conditions reported. (**f**) Results obtained analyzing different quantities of SA genomic DNA pure and mixed with 30 µg of *Triticum turgidum durum* genomic DNA. Mean current values and standard deviations from three replicated measurements are reported.

The processing of raw material to obtain DNA samples is critical for the applicability of the developed magneto-genoassay on *Glycine max* and *Sinapis alba* genomic DNA (Figure 2a), as it determines the quality and yield of the DNA obtained, directly influencing amplification and downstream detection [53].

With the aim of overcoming the issues of DNA fragmentation and reducing the size of genomic DNA into fragments suitable for subsequent detection, we tested a panel of endonuclease enzymes. *Glycine max* genomic extracts were digested non-specifically using DNAse I or in a sequence-specific fashion using restriction enzymes, i.e., TasI, MseI, HindIII, DpnI, and MnlI. The panel of enzymes was selected based on their ability to recognize multiple restriction sites in the Lec1 gene of the *Glycine max* sequence, leaving the region targeted by the magneto-genoassay unaffected. A comparison of the results obtained by performing the genoassay on 5 µg of genomic DNA digested with the selected enzymes (Figure 2b) shows that the restriction enzymes provide better results than non-specific digestion with DNase I. This can be rationalized by considering the possible enzymatic cleavage of the target region by DNAse I, thus hampering the possibility of sandwich complex formation. Comparing the restriction enzymes tested, the best response in terms of signal intensity was obtained with DpnI, which was selected for subsequent studies. To determine the lowest amount of genomic DNA detectable using the magneto-genoassay, *Glycine max* DNA extract was diluted to different quantities ranging from 0.1 to 1 µg and subsequently treated with DpnI (Figure 2c). The magneto-genoassay proved capable of proportionally responding to changes in genomic DNA quantity, detecting down to 0.1 µg of *Glycine max* DNA, which produced significantly different (*p* < 0.05) signals from negative controls. Excellent selectivity was obtained by analyzing 30 µg of genomic DNA extracted from TTD flour after a similar treatment with DpnI, since the obtained signals were not significantly different from the negative control (*p* < 0.05). Furthermore, the effect of TTD genomic DNA on the results obtained was assessed. To this end, 0.1, 0.5 and 1 µg of GM were mixed with 30 µg of TTD before digestion and subsequently analyzed using the magneto-genoassay. The results showed no significant difference (*p* > 0.05) compared to those obtained using pure GM genomic DNA (Figure 2d), thus demonstrating the feasibility of allergen detection in TTD.

As for the detection of *Sinapis alba* genomic DNA, enzymatic digestion of 5 µg of DNA was performed with MnlI, TasI and MseI, since these recognize specific restriction sites present in the target Sin a1 gene. Conversely, HindIII and DpnI were not tested since no restriction site for these endonucleases is present within the targeted gene. In this case, a response was observed only for the sample treated with MnlI, which was selected for testing the magneto-genoassay’s response to different genomic DNA quantities (Figure S5). The lowest DNA level that explored which produced signals different (*p* < 0.05) from wheat was 2 µg, while a dynamic response was observed up to 20 µg (Figure 2e). Also in this case, the analysis of *Triticum turgidum durum* flour samples digested with MnlI did not yield signals statistically different from those of negative controls (*p* > 0.05). Experiments similar to those performed for GM were also carried out to analyze the detection of SA in the presence of TTD, by mixing 1, 5, and 20 µg of SA with 30 µg of TTD before digestion. As in the previous case, no significant difference (*p* > 0.05) was observed in comparison to the results obtained with pure SA genomic DNA (Figure 2f).

These results demonstrate the suitability of magneto-genoassays for the selective detection of genomic DNA from soy and mustard allergens.

## 4. Conclusions

This study outlines a robust and versatile electrochemical magneto-genoassay platform that harnesses the distinctive properties of PNA probes in combination with target-induced bio-orthogonal ligation to achieve highly selective and sensitive allergen DNA detection.

The integration of covalent probe ligation within a sandwich architecture represents a key advancement, effectively overcoming the intrinsic limitations of conventional hybridization-based assays by ensuring assays’ reliability even in the presence of complex food matrices. The superior analytical performance exhibited by functional PNA probes compared to DNA counterparts, particularly with regard to detection limits and linear range, underscores the potential of target-induced bio-orthogonal ligation to enhance nucleic acid-based biosensing strategies. Notably, its successful application to genomic DNA extracted from plant-based food ingredients, combined with excellent selectivity against non-target species, confirms the platform’s applicability in food safety management. It is worth noting that, in addition to its current implementation for the detection of soy and mustard allergens, the modular design of the assay, its compatibility with multichannel and portable electrochemical instrumentation, and the absence of amplification steps ensure its suitability for use in multiplexed, on-site food safety monitoring.

Future developments may focus on expanding the panel of detectable allergens, establishing the methodology as a valuable tool for the food industry for the rapid screening of raw materials, especially when coupled with portable or IoT-enabled devices for decentralized, real-time analysis along the food supply chain.

## 5. Patents

The ligation technology described in the manuscript is part of a patent application by A.M. “Methods For Proximity Mediated Coupling Of A First Agent To A Second Agent” (WO2022079127A1). Other authors have no competing interests to declare.

## Figures and Tables

**Table 1:** Sequences of target DNAs and probes used to target *Glycine max* and *Sinapis alba* allergens using the magneto-genoassays. Capital letters indicate DNA or PNA monomers, small letters indicate L-amino acids, and modifications on the lysine side chains are inserted between brackets. DOP: 4,7-dioxooctanoyl; O: 2-(2-aminoethoxy)ethoxyacetyl (AEEA spacer); Hy: hydrazineacetyl.

Allergen	Probe Nature	Name	Sequence (5′-to-3′ or N-to-C)
*Glycine max*	DNA	GM-T-DNA	CCTTCAACTTCACCTTCTATGCCCCTGACACAAAAAGGCTTGCAG
	DNA	GM-CP-DNA	ATAGAAGGTGAAGTTGAAGG-C_7_-NH_2_
	DNA	GM-SP-DNA	Biotin-TEG-CTGCAAGCCTTTTTGTGTCA
	PNA	GM-CP-11-L	DOP-GAAGTTGAAGG-k(OO)-NH_2_
	PNA	GM-CP-11-Ac	Ac-GAAGTTGAAGG-k(OO)-NH_2_
	PNA	GM-CP-15-L	DOP-GAAGTTGAAGGAAGC-k(OO)-NH_2_
	PNA	GM-SP-11-L	Biotin-OO-GCATAGAAGGT-k(Hy)-NH_2_
	PNA	GM-SP-11-Ac	Biotin-OO-GCATAGAAGGT-k(Ac)-NH_22_
	PNA	GM-SP-15-L	Biotin-OO-AGGGGCATAGAAGGT-k(Hy)-NH_2_
*Sinapis alba*	DNA	SA-T-DNA	AGCTGGGGCAGCAGGGGCAGCAGGGACCGCACCTGCAGCATGTAA
	DNA	SA-CP-DNA	CTGCCCCTGCTGCCCCAGCT-C_7_-NH_2_
	DNA	SA-SP-DNA	Biotin-TEG-TTACATGCTGCAGGTGCGGT
	PNA	SA-CP-11-L	DOP-GCTGCCCCTGC-k(OO)-NH_2_
	PNA	SA-CP-11-Ac	Ac-GCTGCCCCTGC-k(OO)-NH_2_
	PNA	SA-CP-15-L	DOP-GCTGCCCCTGCTGCC-k(OO)-NH_2_
	PNA	SA-SP-11-L	Biotin-OO-GTGCGGTCCCT-k(Hy)-NH_22_
	PNA	SA-SP-11-Ac	Biotin-OO-GTGCGGTCCCT-k(Ac)-NH_22_
	PNA	SA-SP-15-L	Biotin-OO-GCAGGTGCGGTCCCT-k(Hy)-NH_2_

**Table 2:** Performance comparison of magneto-genoassay based on ligation-capable PNA probes or DNA probes for the detection of *Glycine max* and *Sinapis alba* allergen DNA.

	DNA-Based Magneto-Genoassay	PNA-Based Magneto-Genoassay	DNA-Based Magneto-Genoassay	PNA-Based Magneto-Genoassay
	*Glycine max*		*Sinapis alba*	
	(pM)			
Linear response range	300–1000	50–1000	200–1000	100–1000
LOD	33	16	22	19
LOQ	110	48	156	59

**Table 3:** Comparison of the proposed method with the other reported electrochemical genoassays for food allergen detection.

Analytical Approach	Target	LOD	Analysis Time (Minutes)	Amplification	Reference
Sandwich scaffold assembled on magnetic microbeads with HRP enzyme tag	Cor a 9 (Hazelnut)	0.72 pM	Amplification: 40 Detection: 15	Yes	[49]
DNA/RNA heteroduplex sandwich assembled on MBs with HRP-antibody labelling	Sin a 1 (Mustard)	3 pM	Detection: 75	No	[24]
Sandwich scaffold assembled on straptavidinated MBs with ALP enzyme tag	Cor a 1.04 (Hazelnut)	200 pM	Amplification: 55 Detection: 35	Yes	[50]
Sandwich scaffold assembled on a gold SPE enzymatically tagged with Anti-FITC-HRP	Gliadin	300 pM	Amplification: 42 Detection: 150	Yes	[51]
Multiplex sandwich scaffold assembled on MB with ALP and HRP enzyme tags	Roundup Ready^®^ soybean	8.2 pg	Amplification: 60 Detection: 90	Yes	[52]
PNA-based sandwich scaffold favoured by ligation, assembled on MB with ALP enzyme tag	Mustard and soybean	16 pM (soybean) 19 pM (mustard)	Detection: 75	No	This study

## Data Availability

All data generated or analyzed during this study are included in this published article and its Supplementary Information file.

## Supplementary Materials

The following supporting information can be downloaded at: https://www.mdpi.com/article/10.3390/bios16060340/s1, Figure S1: Effect of DNA probes concentration; Figure S2: Calibration curves obtained using PNA probes; Figure S3: Calibration curves obtained using DNA probes; Figure S4: Cross-reactivity studies; Figure S5: Effect of digestion of *Sinapis alba* genomic DNA with different restriction enzymes; Figure S6: alignment of designed targeting regions, Sinapis alba Sin a I sequence and Brassica Napus storage proteins sequences; Figures S7–S18: HPLC-UV-MS chromatogram of purified PNAs. Table S1. PNA sequences used in this study. Reference [54] is cited in the Supplementary Materials.
