# Supplementary material for: Ligation-Driven Electrochemical Magneto-Genoassay Platform Based on PNA Probes for the Multiple Detection of Soy and Mustard DNA in Wheat Flour
Source: Biosensors (Basel). 2026 Jun 16;16(6):340. doi: 10.3390/bios16060340 (PMC13297038; doi:10.3390/bios16060340)
Supplement: Supplementary file 1 [file biosensors-16-00340-s001.zip › biosensors-4302837-supplementary.pdf]

Supporting Information

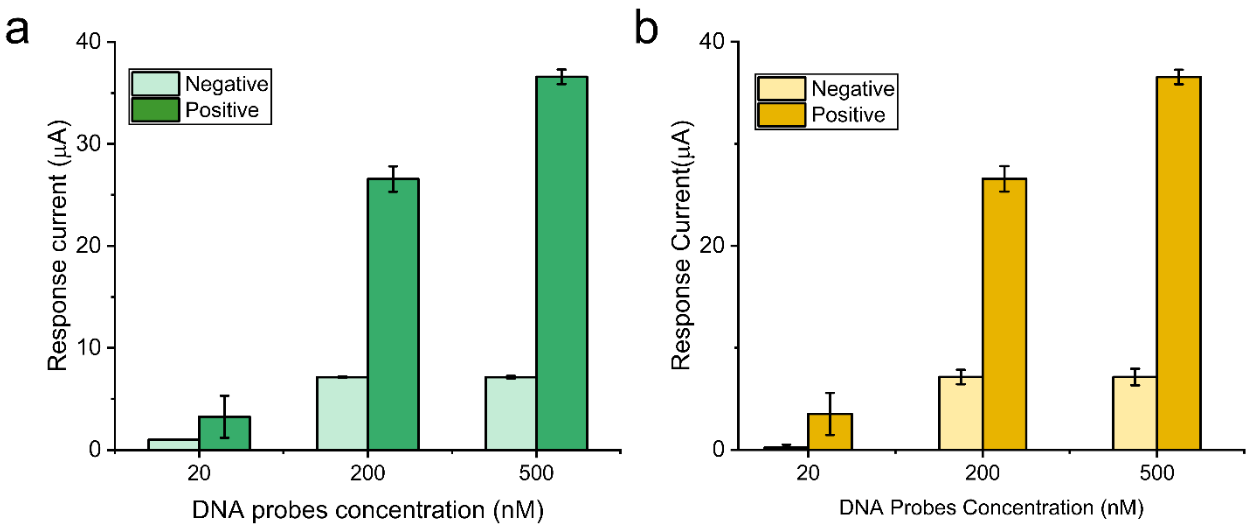

Figure S1. Effect of DNA capture and signalling probe concentrations on the detection of a) *Glycine max* and b) *Sinapis alba*. Mean current values and standard deviations from three replicated measurements are reported.

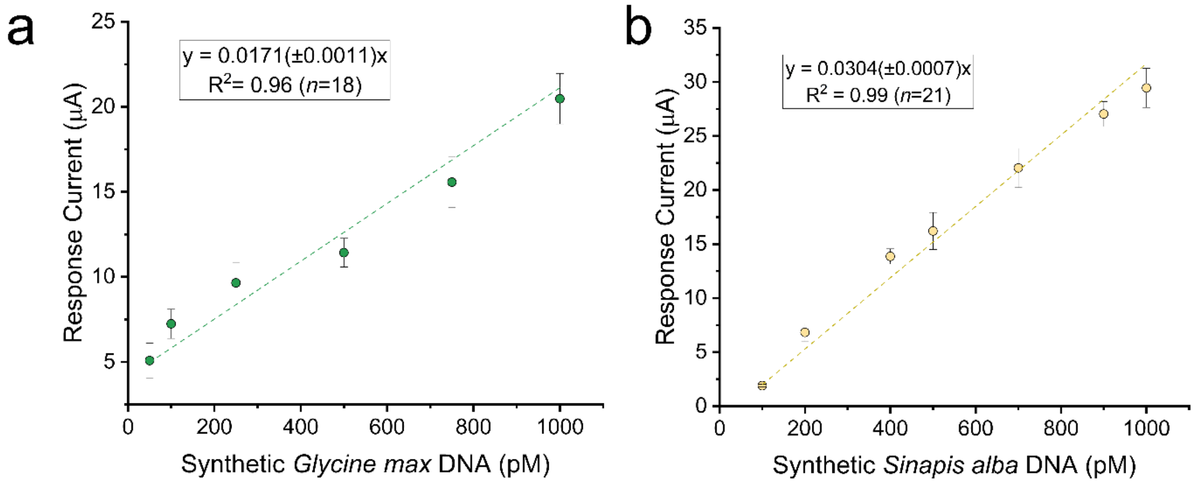

Figure S2. Calibration curves obtained on synthetic DNA using the magneto-genoassays based on ligation-capable PNA probes a) *Glycine max* and b) *Sinapis alba*.

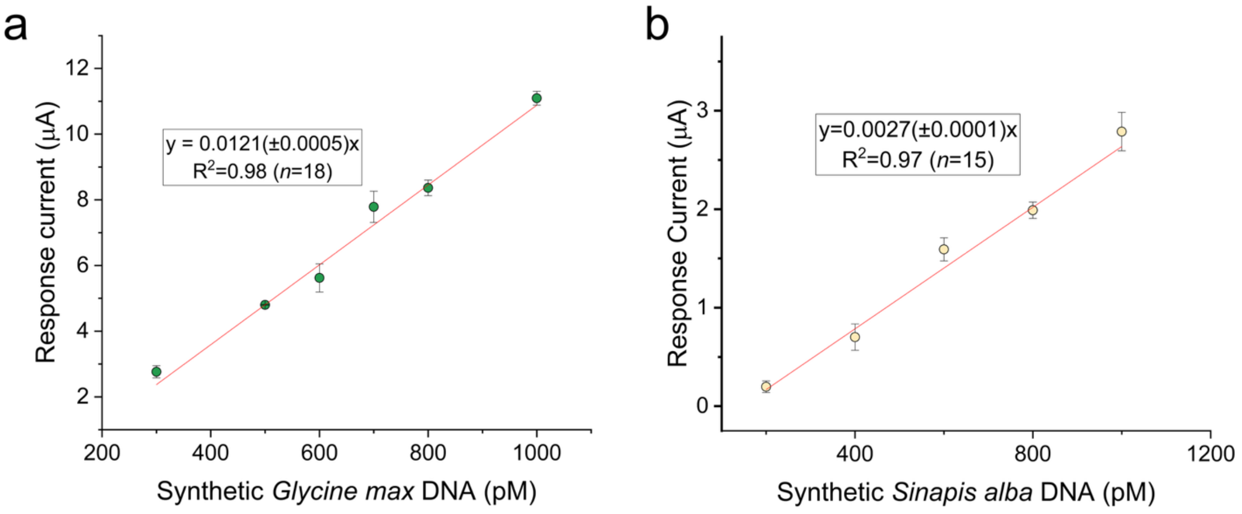

Figure S3. Calibration curves obtained on synthetic DNA using the magneto-genoassays based on DNA probes t a) *Glycine max* and b) *Sinapis alba*.

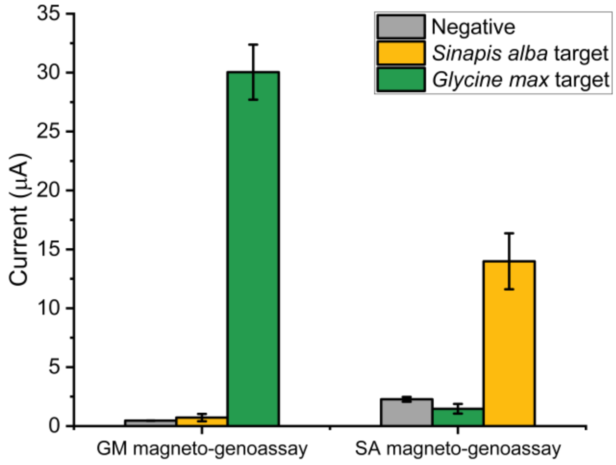

Figure S4. Cross-reactivity responses assessed using the PNA-based magneto-genoassays for the detection of SA and GM synthetic target DNA. Mean current values and standard deviations from three replicated measurements are reported.

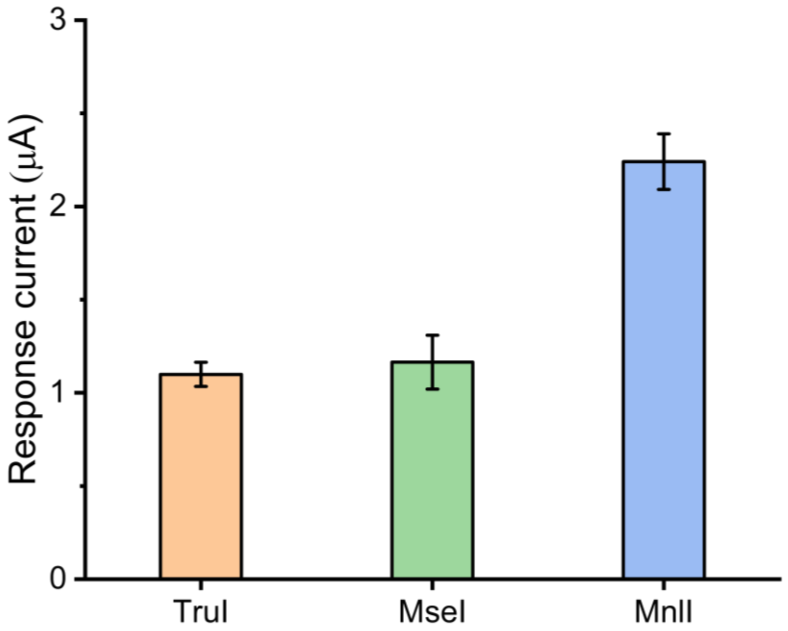

Figure S5. Test results obtained analysing *Sinapis alba* genomic DNA digested with different endonuclease enzymes.

General information

All reagents were purchased from Sigma-Aldrich, Iris Biotech, PolyOrg, Merck, and TCI Europe and used without further purification. Dry DMF was stored over 4 Å molecular sieves.

HPLC-UV was used for the purification of PNA. The Agilent 1260 Infinity II instrument equipped with a Diode Array Detector (DAD) . Separation was performed using the Phenomenex Luna C18(2) 100 Å, 5 µm, 250 mm x 10 mm column at 40°C or 60°C, for 11mer and 15mer probes respectively, with the following solvent systems: (A): 0.1% TFA in H_2O and (B) 0.1% TFA in MeCN. Gradient elution was carried out at a flow-rate of 4 mL/min starting with 100% solvent A for 1.0 minute, then the composition was linearly changed to 65% A and 35% B over 21 minutes (1.0–22.0 min), followed by a ramp to 100% B over 0.5 minutes (22.0–22.5 min) and finally held at 100% B for 5 minutes. The system was then returned to 100% A over 0.5 min (27.5–28.0 min) and maintained at 100% A for 9 minutes.

HPLC-UV-MS analysis of PNA probes was performed using the Agilent 1260 Infinity II system equipped with a DAD and the Agilent Jet Stream-interface coupled to Agilent InfinityLab LC/MSD XT. Separation was performed using the Waters Acquity UPLC BEH 300 (50 × 2.1 mm, 1.7 µm, C18) column with the following solvent system: (A): 0.2% HCOOH in H_2O and (B) 0.2% HCOOH in MeCN. Gradient elution was carried out at a flow-rate of 0.35 mL/min starting with 100% solvent A for 0.2 minute, then the composition was linearly changed to 50% A and 50% B over 8 minutes (0.2–8.2 min), then ramped to 100% B over 0.1 minutes (8.2–8.3 min) and held at 100% B for 2 minutes. The system was then returned to 100% A over 0.1 minutes (10.3–10.4 min) and maintained at 100% A for 5 minutes. Full scan acquisition was performed from 150 to 1500 amu.

PNA synthesis protocols

Automatic solid phase synthesis using a MultiSynTech GmbH Syro I peptide synthesizer: PNA monomers and HBTU were dissolved in dry DMF at concentrations of 0.1 M and 0.47 M, respectively; DIPEA was diluted in dry DMF at concentration of 0.40 M. After swelling in dichloromethane (DCM) it was followed a cyclic

procedure: a) deprotection with 20% piperidine in DMF (2x 8 min), b) coupling with PNA monomer (5 equivalents), HBTU (4.7 equivalents), DIPEA (10 equivalents) in dry DMF (2x 30 min), c) capping with acetic anhydride/DIPEA/DMF 5:6:95 (2x 1 min).

Manual solid phase synthesis: a) deprotection with 20% piperidine in DMF (2x 8 min) or Mtt (HFIP/DCM 1:1, containing 90 mM HOBt, 4x 3 minutes), b) coupling with 3-(5-methyfuran-2-yl-)propionate, Fmoc-AEEA-OH, Boc-AEEA-OH, Tri-Boc-hydrazinoacetic acid or biotin (5 equivalents at c = 0.05 M), HBTU (4.9 equivalents at c = 0.049 M), DIPEA 10 equivalents, c = 0.1 M) in dry DMF (2 minutes activation followed by 40-90 minutes as reaction time), c) capping with acetic anhydride/DIPEA/DMF 5:6:95, (2x 1 minutes), d) washing with 5% DIPEA in DMF to remove traces of acetic anhydride (2x 2 min). PNAs were assembled by automated solid-phase synthesis, except GM-CP-15-L and SA-SP-15-L, in which the residues from monomer n11 were coupled manually. All couplings involving non-standard PNA-building blocks were performed manually. For the signalling probe, the N-terminal Fmoc-AEEA-OH spacers were introduced using a coupling time of 40 min, followed by biotin coupling time of 90 min, while the α-nucleophile was introduced at the C-terminus using a 90 min coupling time, after Mtt removal. For the capture probe, 3-(5-methylfuran-2-yl)propanoic acid was introduced at the N-terminus using a 90 min coupling; C-terminal spacers were subsequently installed, after Mtt removal, using a coupling time of 90 min.

PNAs were cleaved from the solid support using a TFA/m-cresol/thioanisole (8:1:1 v/v) cleavage cocktail and then precipitated in diethyl ether. Probes were then dissolved in water and purified by RP-HPLC. PNA purity and identity were assessed by HPLC-UV-MS and yield was determined after solubilization in mQ and calculating their concentration from the absorbance at 260 nm using Lambert-Beer's law, assuming an additive contribution of all nucleobases.

Table S1. PNA sequences used in this study. Capital letters indicate PNA monomers, small letters indicate L-amino acids, and modifications on the lysine side chains are inserted inside brackets. DOP: 4,7-dioxooctanoyl; O: 2-(2-aminoethoxy)ethoxyacetyl (AEEA spacer); Biot: Biotinyl; Hy: hydrazineacetyl.

Probe name	Sequence (N to C)	MW (g/ mol)
GM-CP-11-L	DOP-GAAGTTGAAGG-k(OO)-NH_2	3679.6
GM-CP-11-Ac	Ac-GAAGTTGAAGG-k(OO)-NH_2	3567.5
GM-CP-15-L	DOP-GAAGTTGAAGGAAGC-k(OO)-NH_2	4772.7
GM-SP-11-L	Biot-OO-GCATAGAAGGT-k(Hy)-NH_2	3783.8
GM-SP-11-Ac	Biot-OO-GCATAGAAGGT-k(Ac)-NH_2	3753.8
GM-SP-15-L	Biot-OO-AGGGGCATAGAAGGT-k(Hy)-NH_2	4932.9
SA-CP-11-L	DOP-GCTGCCCCTGC-k(OO)-NH_2	3503.5
SA-CP-11-Ac	Ac-GCTGCCCCTGC-k(OO)-NH_2	3391.4
SA-CP-15-L	DOP-GCTGCCCCTGCTGCC-k(OO)-NH_2	4563.6
SA-SP-11-L	Biot-OO-GTGCGGTCCCT-k(Hy)-NH_2	3702.7
SA-SP-11-Ac	Biot-OO-GTGCGGTCCCT-k(Ac)-NH_2	3672.7
SA-SP-15-L	Biot-OO-GCAGGTGCGGTCCCT-k(Hy)-NH_2	4811.8

GM-CP-11-L: 2.7%; t_r: 4.32 min; ε = 130500 $M^{-1}cm^{-1}$; ESI-MS: MW calcd 3679.6 [M]; m/z found 1227.5 $[M+3H]^{3+}$, 920.9 $[M+4H]^{4+}$, 736.9 $[M+5H]^{5+}$, 733.4* $[M-H_2O+5H]^{5+}$, 614.3 $[M+6H]^{6+}$, 611.3* $[M-H_2O+6H]^{6+}$, 526.7 $[M+7H]^{7+}$, 524.1* $[M-H_2O+7H]^{7+}$. **GM-CP-11-Ac**: 5.7%; t_r: 4.06 min; ε = 130500 $M^{-1}cm^{-1}$; ESI-MS: MW calcd 3567.5 [M]; m/z found 1190.1 $[M+3H]^{3+}$, 892.9 $[M+4H]^{4+}$, 714.5 $[M+5H]^{5+}$, 595.6 $[M+6H]^{6+}$, 510.7 $[M+7H]^{7+}$. **GM-CP-15-L:** 0.5%; t_r: 4.10 min; ε = 176200 $M^{-1}cm^{-1}$; ESI-MS: MW calcd 4772.7 [M]; m/z found 1194.2 $[M+4H]^{4+}$, 955.6 $[M+5H]^{5+}$, 796.5

$[M+6H]^{6+}$, 793.5* $[M-H_2O+6H]^{6+}$, 682.9 $[M+7H]^{7+}$, 680.3* $[M-H_2O+7H]^{7+}$, 597.7 $[M+8H]^{8+}$, 595.4* $[M-H_2O+8H]^{8+}$, 531.4 $[M+9H]^{9+}$, 529.4* $[M-H_2O+9H]^{9+}$. **GM-SP-11-L**: 7.4%; t_r: 4.50 min; ε = 125400 $M^{-1}cm^{-1}$; ESI-MS: MW calcd 3783.8 [M]; m/z found 1262.2 $[M+3H]^{3+}$, 946.9 $[M+4H]^{4+}$, 757.8 $[M+5H]^{5+}$, 631.7 $[M+6H]^{6+}$, 541.6 $[M+7H]^{7+}$. **GM-SP-11-Ac:** 7.6%; tr: 4.78 min; ε = 125400 $M^{-1}cm^{-1}$; ESI-MS: m/z calcd 3753.8 [M]; m/z found 1252.1 $[M+3H]^{3+}$, 939.4 $[M+4H]^{4+}$, 751.8 $[M+5H]^{5+}$, 626.7 $[M+6H]^{6+}$. **GM-SP-15-L:** 1.0%; t_r: 4.32 min; ε = 174200 $M^{-1}cm^{-1}$; ESI-MS: MW calcd 4932.9 [M]; m/z found 1234.3 $[M+4H]^{4+}$, 987.7 $[M+5H]^{5+}$, 823.2 $[M+6H]^{6+}$, 705.8 $[M+7H]^{7+}$, 617.7 $[M+8H]^{8+}$. **SA-CP-11-L**: 10.4%; t_r: 4.03 min; ε = 91900 $M^{-1}cm^{-1}$; ESI-MS: MW calcd 3503.5 [M]; m/z found 1168.8 $[M+3H]^{3+}$, 876.8 $[M+4H]^{4+}$, 701.7 $[M+5H]^{5+}$, 698.1* $[M-H_2O+5H]^{5+}$, 584.9 $[M+6H]^{6+}$, 582.0* $[M-H_2O+6H]^{6+}$, 501.5 $[M+7H]^{7+}$, 498.9* $[M-H_2O+7H]^{7+}$. **SA-CP-11-Ac**: 15.2%; t_r: 3.85 min; ε = 91900 $M^{-1}cm^{-1}$; ESI-MS: MW calcd 3391.4 [M]; m/z found 1131.4 $[M+3H]^{3+}$, 848.8 $[M+4H]^{4+}$, 679.3 $[M+5H]^{5+}$, 566.3 $[M+6H]^{6+}$, 485.5 $[M+7H]^{7+}$. **SA-CP-15-L:** 7.6%; t_r: 4.19 min; ε = 125400 $M^{-1}cm^{-1}$; ESI-MS: MW calcd 4563.6 [M]; m/z found 1141.9 $[M+4H]^{4+}$, 913.8 $[M+5H]^{5+}$, 761.6 $[M+6H]^{6+}$, 758.7* $[M-H_2O+6H]^{6+}$, 653.0 $[M+7H]^{7+}$, 650.4* $[M-H_2O+7H]^{7+}$, 571.5 $[M+8H]^{8+}$, 569.3* $[M-H_2O+8H]^{8+}$, 508.1 $[M+9H]^{9+}$, 506.1* $[M-H_2O+9H]^{9+}$. **SA-SP-11-L**: 11.6%; t_r: 4.5 min; ε = 99000 $M^{-1}cm^{-1}$; ESI-MS: MW calcd 3702.7 [M]; m/z found 1235.1 $[M+3H]^{3+}$, 926.6 $[M+4H]^{4+}$, 741.5 $[M+5H]^{5+}$, 618.2 $[M+6H]^{6+}$, 530.0 $[M+7H]^{7+}$. **SA-SP-11-Ac**: 9.5%; t_r: 4.83 min; ε = 99000 $M^{-1}cm^{-1}$; ESI-MS: MW calcd 3672.7 [M]; m/z found 1225.3 $[M+3H]^{3+}$, 919.2 $[M+4H]^{4+}$, 735.6 $[M+5H]^{5+}$, 613.2 $[M+6H]^{6+}$. **SA-SP-15-L:** 3.9%; t_r: 4.54 min; ε = 142700 $M^{-1}cm^{-1}$; ESI-MS: MW calcd 4811.8 [M]; m/z found 1204.0 $[M+4H]^{4+}$, 963.4 $[M+5H]^{5+}$, 803.0 $[M+6H]^{6+}$, 688.5 $[M+7H]^{7+}$, 602.6 $[M+8H]^{8+}$. * ESI-MS induced dehydration of the DOP moiety.

Mustard target region identification

All DNA sequences were downloaded from GenBank. DNA sequence encoding for Sinapis Alba major allergen Sin a I (Seq.ID: X91799.1)[1], a 2-S albumin storage protein, and the major allergen of yellow mustard, were aligned to available Brassica Napus Napin storage protein genes (napin ID: J02782.1; napA ID: J02798.1, napB ID: X14492.1; BngNAP1 ID: X17542.1; BnNa ID: M64633.1) using Gene Doc. Region showing the highest mismatch degree between *Sin a I* and other genes was selected as target region for the design of the detection probes.

```
PNA target region        AACAGCTGGGGCAGCAGGGGCAGCAGGGACCGCACCTGCAGCATGTAATT
DNA target region        AACAGCTGGGGCAGCAGGGGCAGCAGGGACCGCACCTGCAGCATGTAATT
S.Alba Sin a I    : agacaacagctggggcagcaggggcagcagggaccgcacctgcagcatgtaattagccgt
B.napus_napin     : cgacaacaacagggacaacaaa---tgcagggacagcagatgcagcaaatggtgagccgt
B.Napus_napA      : ---caacaacagggacaaca------gcaagga------aagcagcaaatggtgagccgt
B.Napus_napB      : ---caacaacagggacaaca------gcaagga------aagcagcaaatggtgagccgt
B.Napus_BngNAP1   : cgacaacagcaaggaca---------gcagggacagcagctgcagcaaatggtgagccgt
B.Napus_BnNa      : cgacaacaacagggacaacaaa---tgcagggacagcagatgcagcaaatggtgagccgt
```

Figure S6. alignment of designed targeting regions, Sinapis Alba Sin a I sequence and Brassica Napus storage proteins sequences (focus). Capture probes targeting regions are highlighted in grey; reported probes targeting regions are highlighted in sky blue. Matching bases are highlighted in green, mismatched bases are highlighted in yellow (A<>G or C<>T) or red (purine<>pirimidine).

Melting temperatures prediction

Melting temperatures (T_m) for PNA-DNA duplexes were calculated using https://pnabio.com/pna-tool/. DNA-DNA duplex T_m values were calculated using https://ita.promega.com/resources/tools/biomath/tm-calculator/ with an oligonucleotide concentration of 4000 nM (same concentration used for PNAs), $[Na^+]$ = 140 mM, and $[K^+]$ = 0 mM.

HPLC-MS chromatograms of pure PNAs

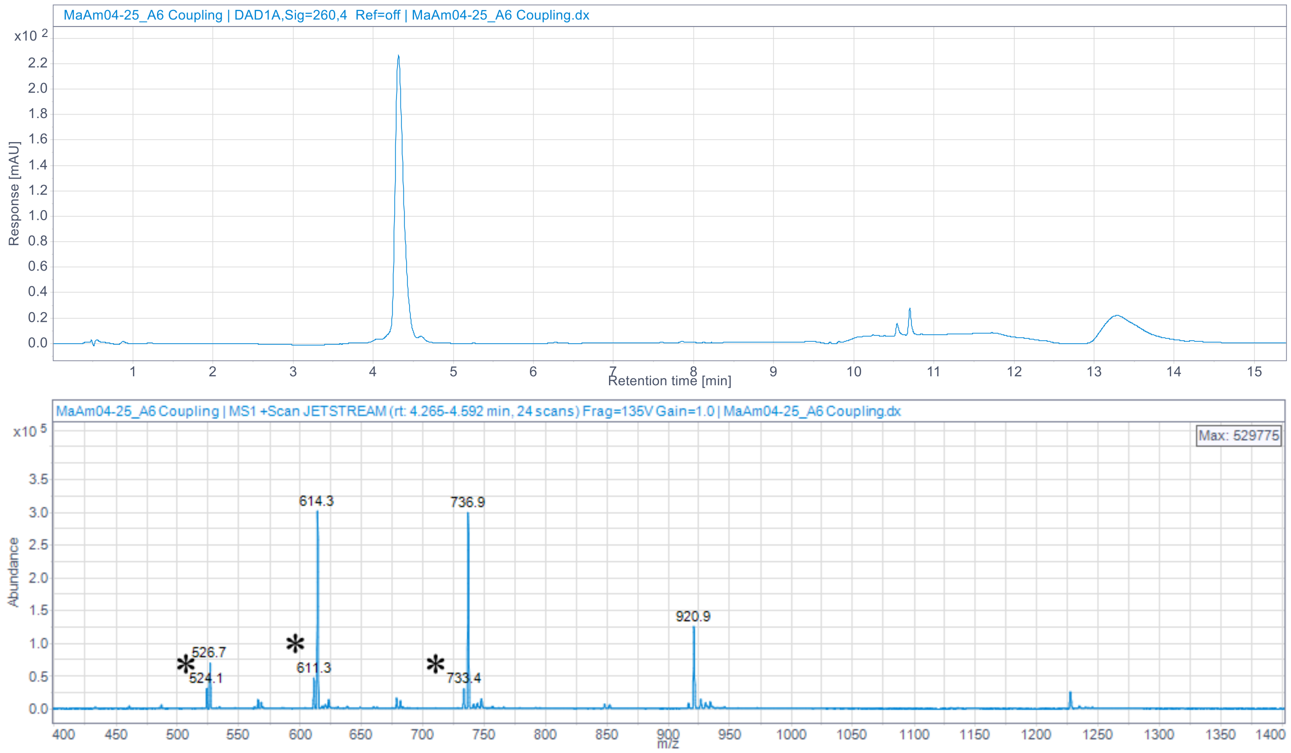

Figure S7. HPLC-UV-MS chromatogram of purified **GM-CP-11-L**. HPLC-UV trace at 260 nm (top) and MS spectrum of the corresponding peak at t_r: 4.32 min (bottom). Calcd MW: 3679.68 Da (Target PNA) and MW: 3661.68 Da (Target PNA dehydrated, loss of water molecule from DOP moiety during ionization).

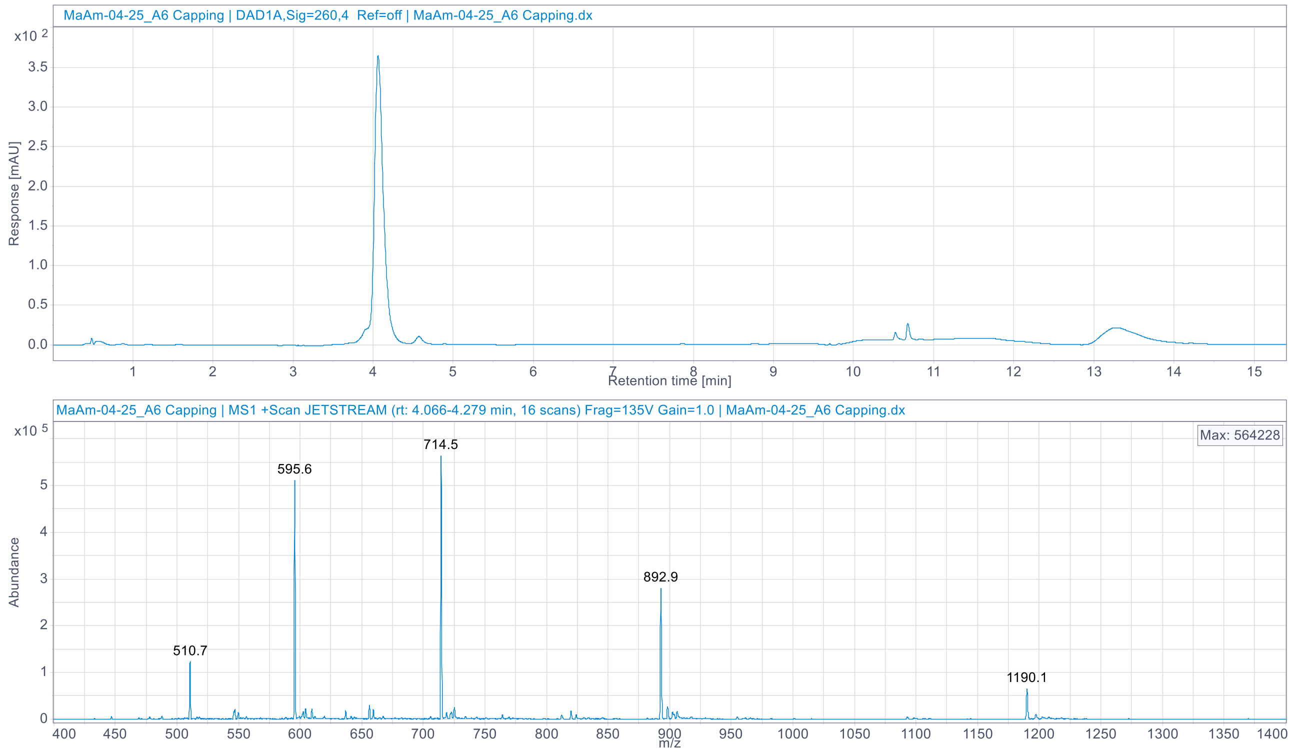

Figure S8. HPLC-UV-MS chromatogram of purified **GM-CP-11-Ac**. HPLC-UV trace at 260 nm (top) and MS spectrum of the corresponding peak at t_r: 4.06 min (bottom). Calcd MW: 3567.62 Da.

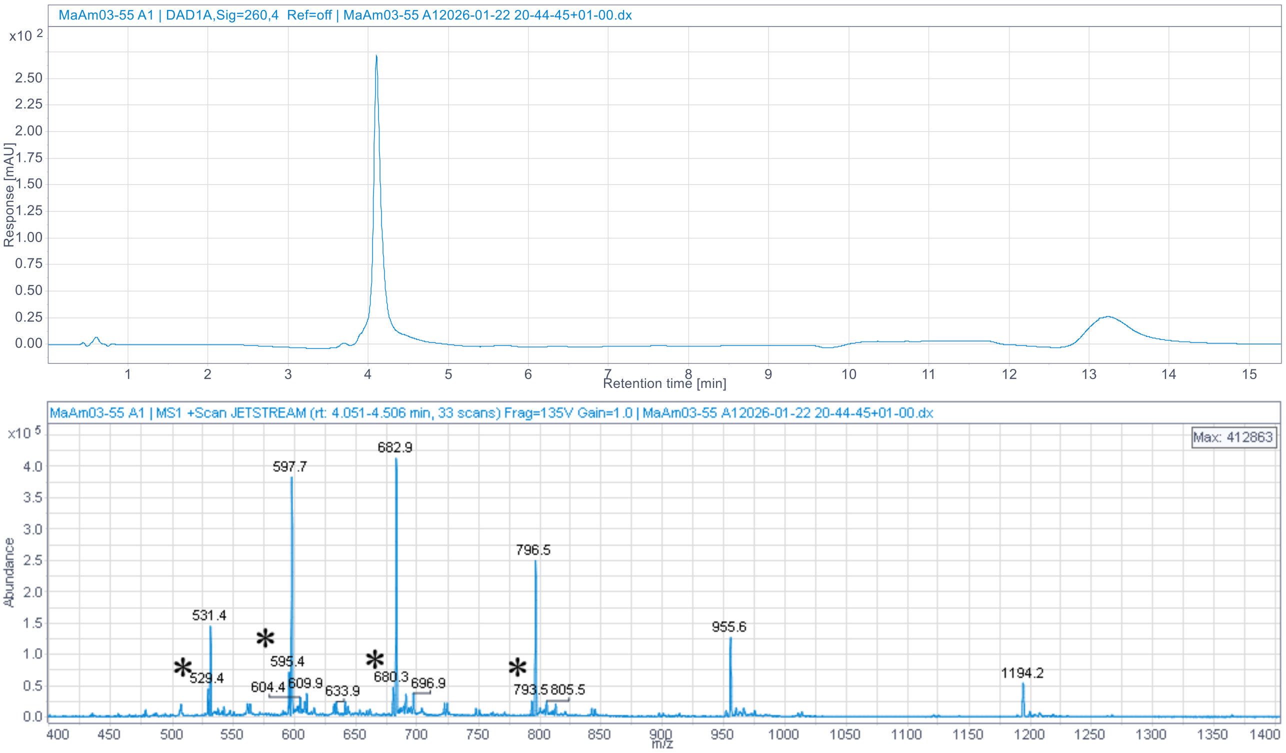

Figure S9. HPLC-UV-MS chromatogram of purified **GM-CP-15-L**. HPLC-UV trace at 260 nm (top) and MS spectrum of the corresponding peak at t_r: 4.10 min (bottom). Calcd MW: 4772.70 Da (Target PNA), and MW: 4754.72 Da (Target PNA dehydrated, loss of water molecule from DOP moiety during ionization).

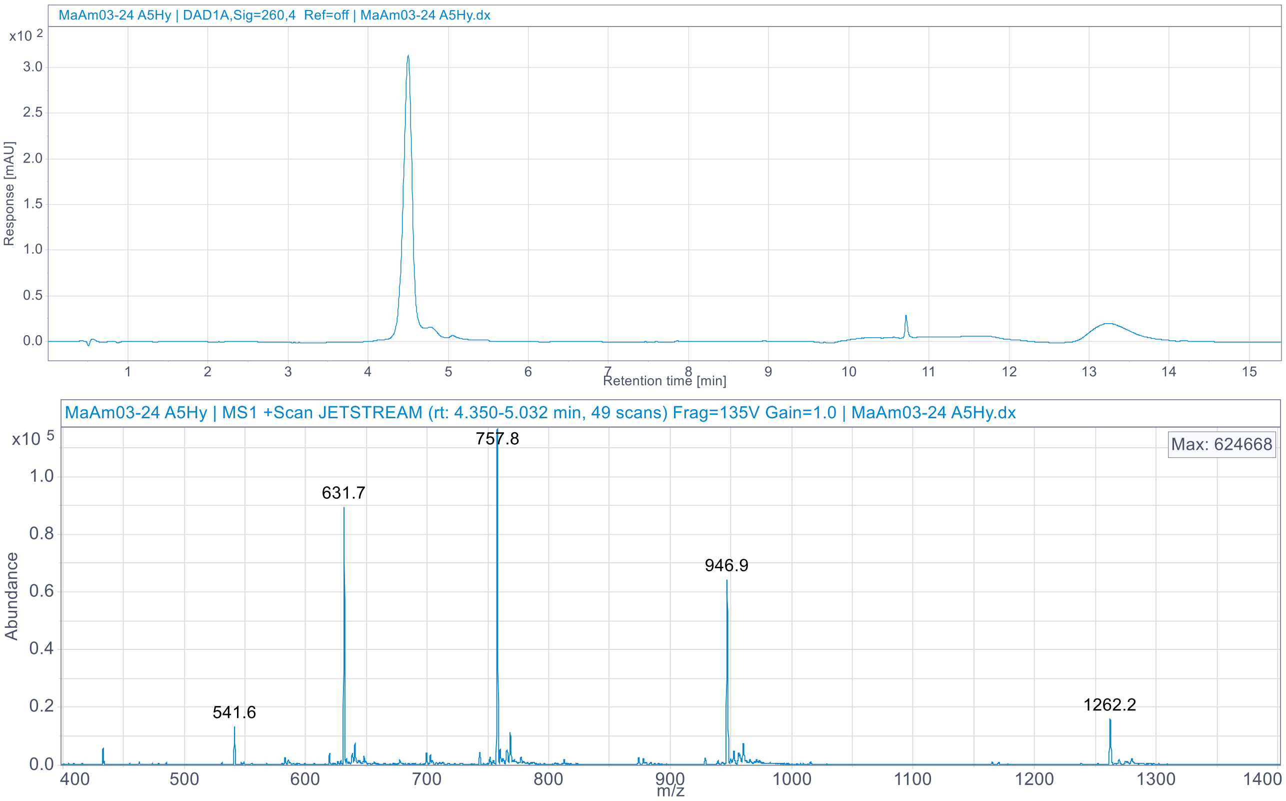

Figure S10. HPLC-UV-MS chromatogram of purified **GM-SP-11-L**. HPLC-UV trace at 260 nm (top) and MS spectrum of the corresponding peak at t_r: 4.50 min (bottom). Calcd MW: 3783.9 Da.

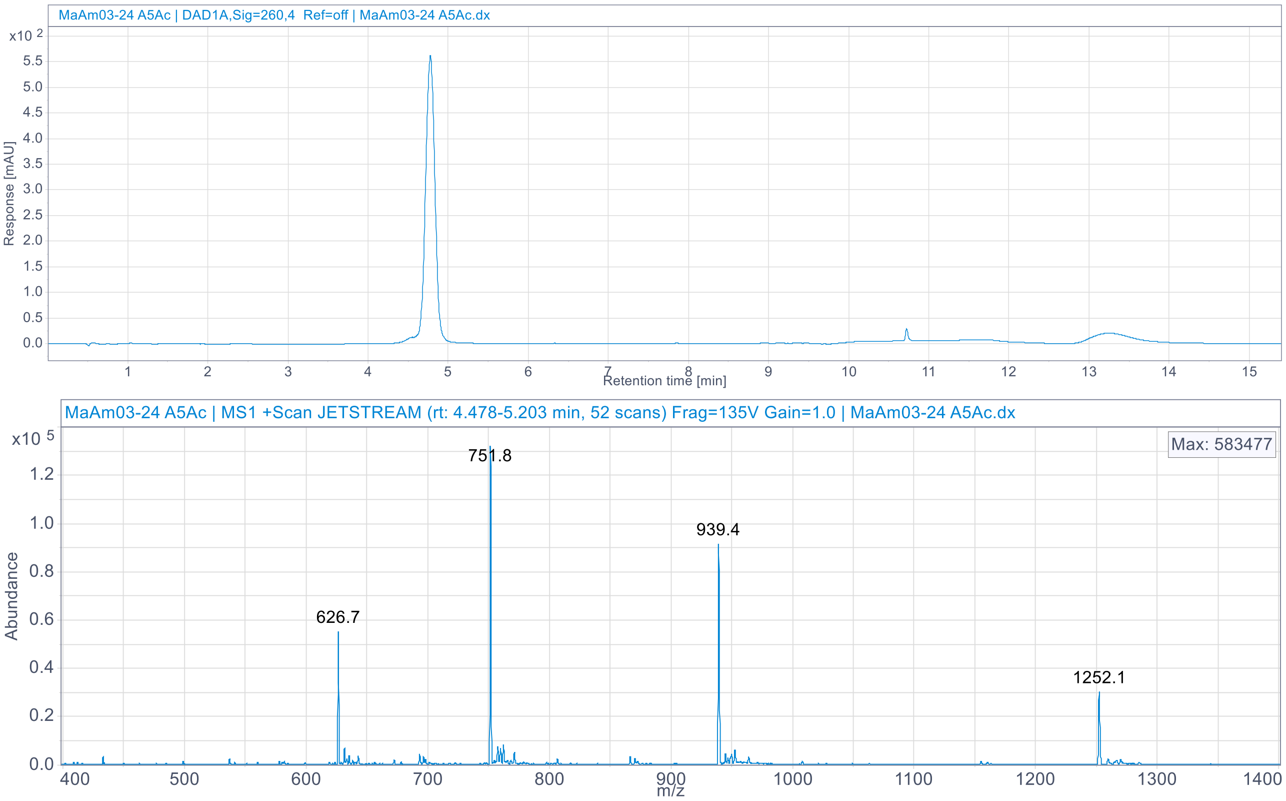

Figure S11. HPLC-UV-MS chromatogram of purified **GM-SP-11-Ac**. HPLC-UV trace at 260 nm (top) and MS spectrum of the corresponding peak at t_r: 4.78 min (bottom). Calcd MW: 3753.8 Da.

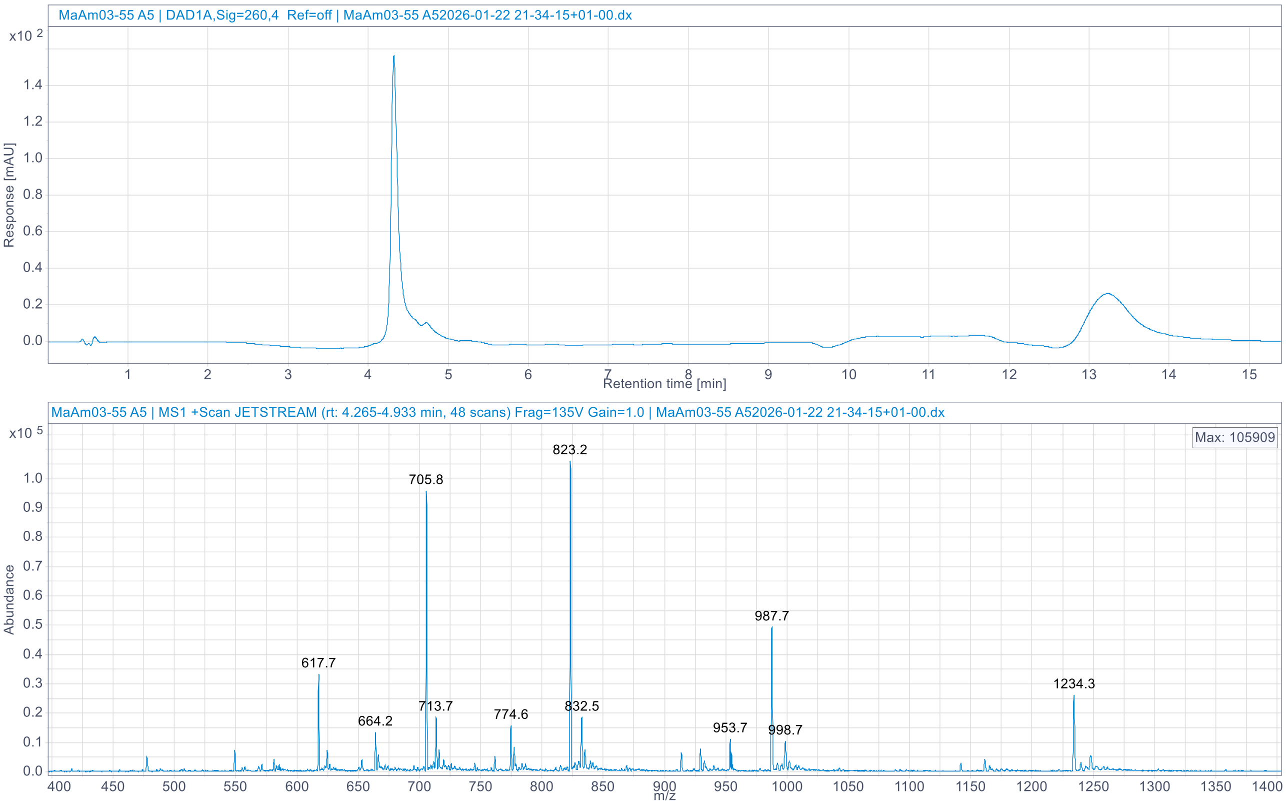

Figure S12. HPLC-UV-MS chromatogram of purified **GM-SP-15-L**. HPLC-UV trace at 260 nm (top) and MS spectrum of the corresponding peak at t_r: 4.32 min (bottom). Calcd MW: 4932.89 Da.

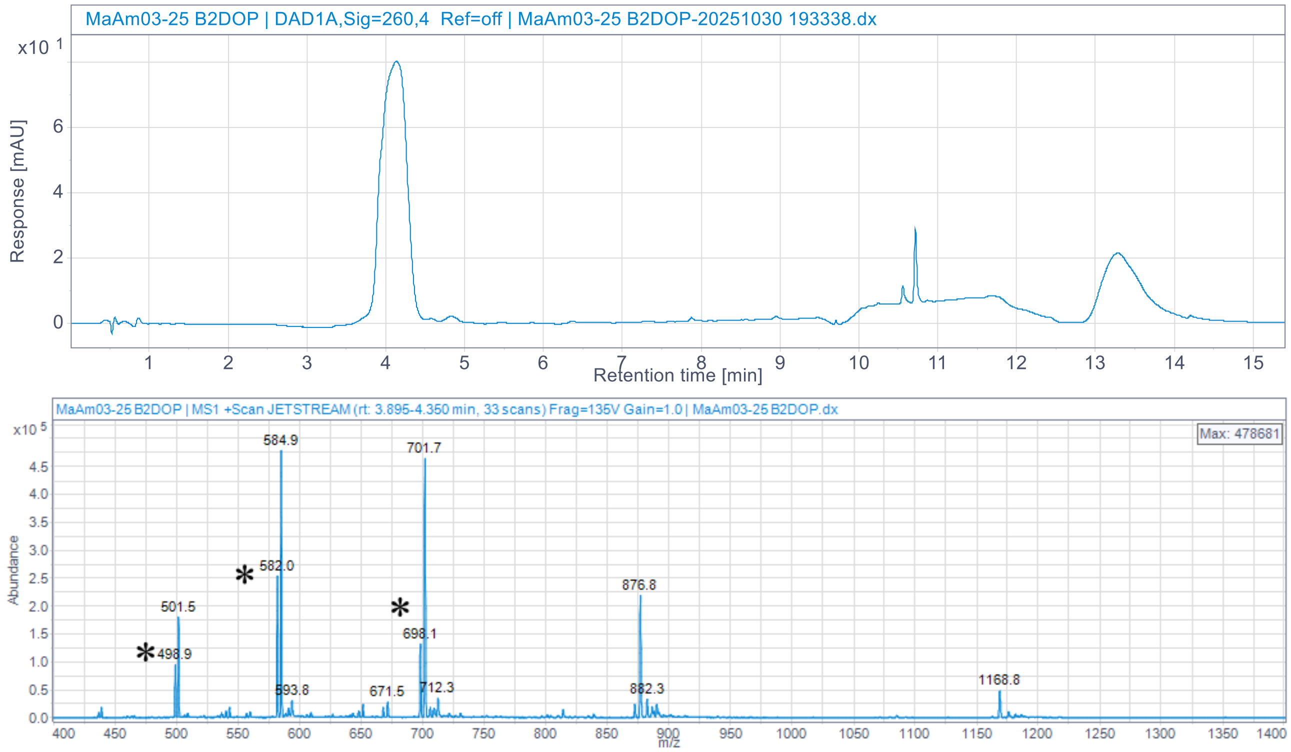

Figure S13. HPLC-UV-MS chromatogram of purified **SA-CP-11-L**. HPLC-UV trace at 260 nm (top) and MS spectrum of the corresponding peak at t_r: 4.03 min (bottom). Calcd MW: 3503.59 Da (Target PNA) and MW: 3485.59 Da (Target PNA dehydrated, loss of water molecule from DOP moiety during ionization).

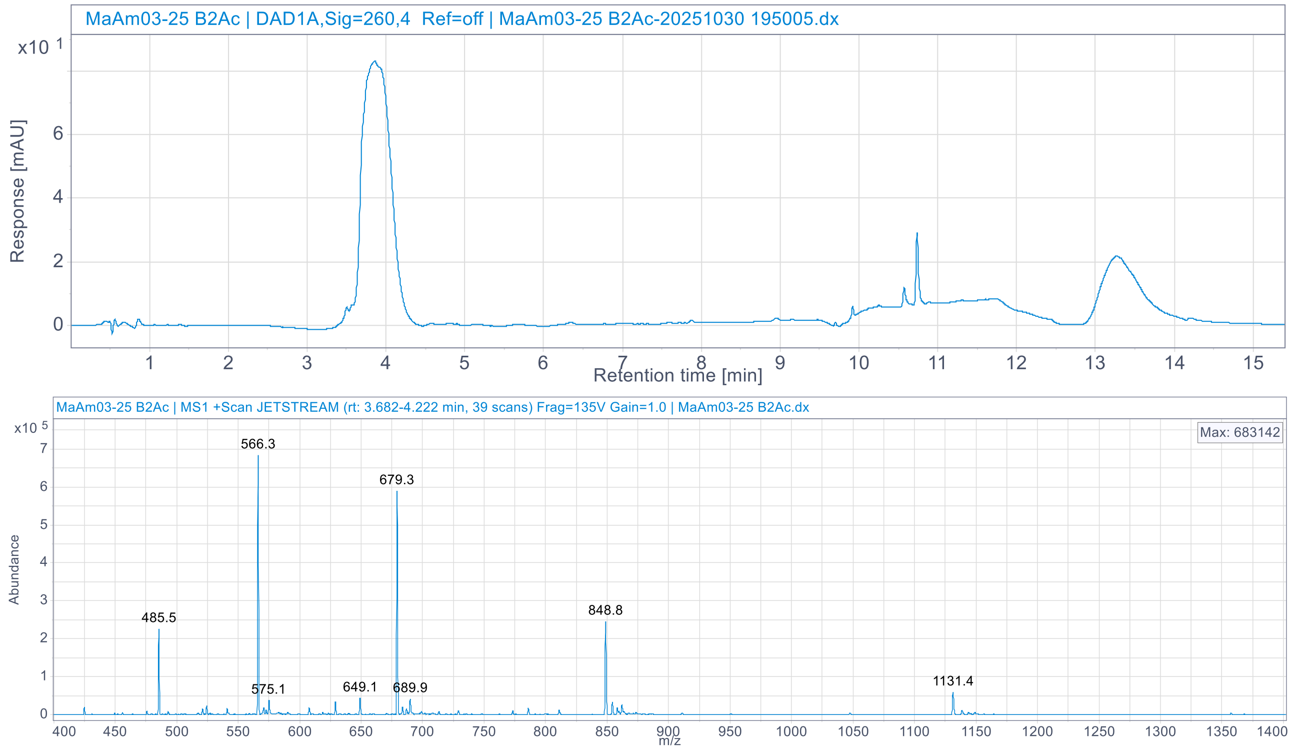

Figure S14. HPLC-UV-MS chromatogram of purified **SA-CP-11-Ac**. HPLC-UV trace at 260 nm (top) and MS spectrum of the corresponding peak at t_r: 3.85 min (bottom). Calcd MW: 3391.48 Da.

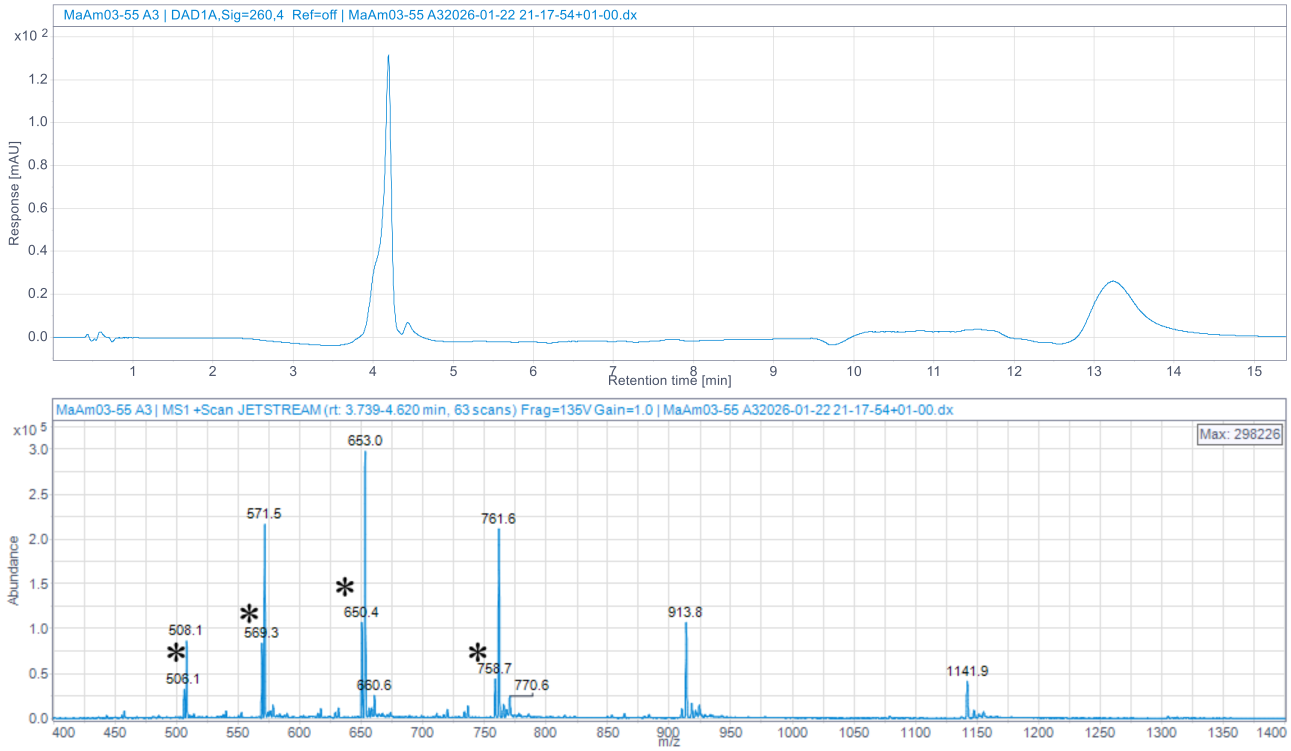

Figure S15. HPLC-UV-MS chromatogram of purified **SA-CP-15 L**. HPLC-UV trace at 260 nm (top) and MS spectrum of the corresponding peak at t_r: 4.19 min (bottom). Calcd MW: 4564.05 Da (Target PNA), and MW: 4546.05 Da (Target PNA dehydrated, loss of water molecule from DOP moiety during ionization).

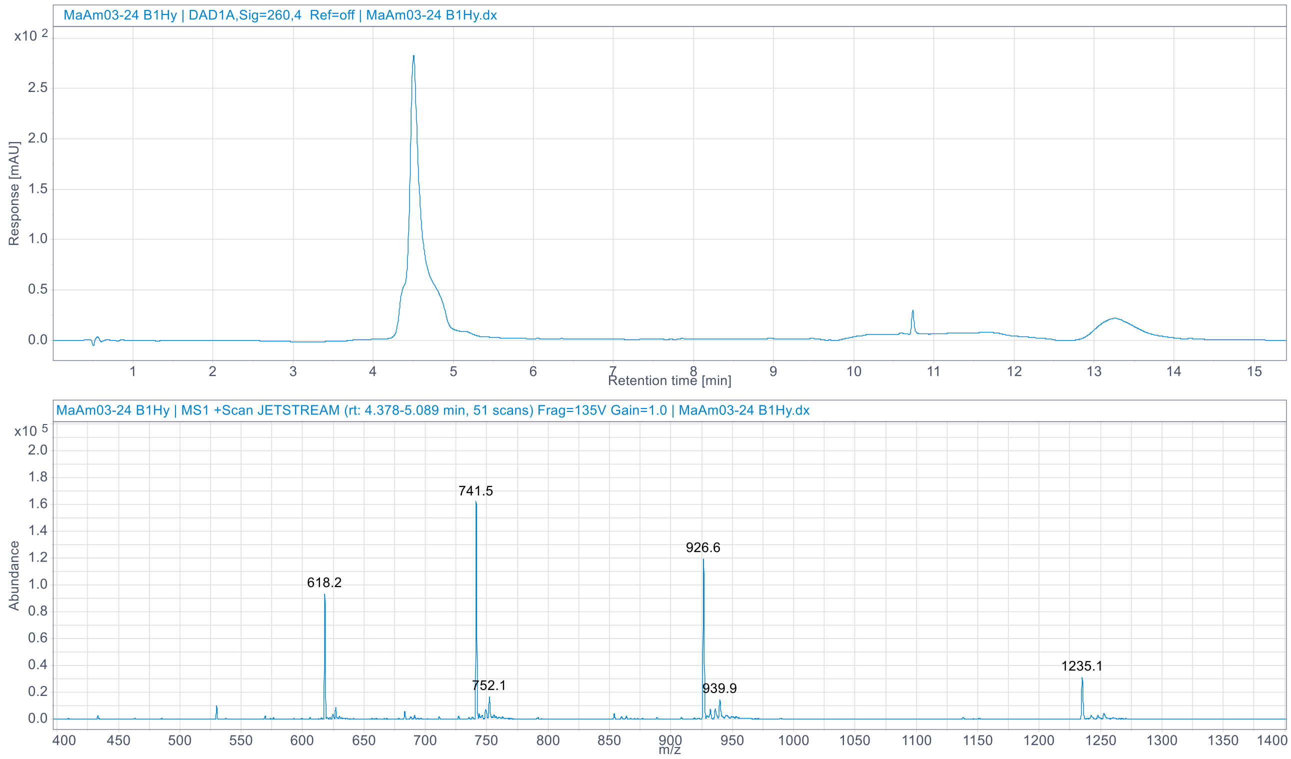

Figure S16. HPLC-UV-MS chromatogram of purified **SA-SP-11-L**. HPLC-UV trace at 260 nm (top) and MS spectrum of the corresponding peak at t_r: 4.5 min (bottom). Calcd MW: 3702.74 Da.

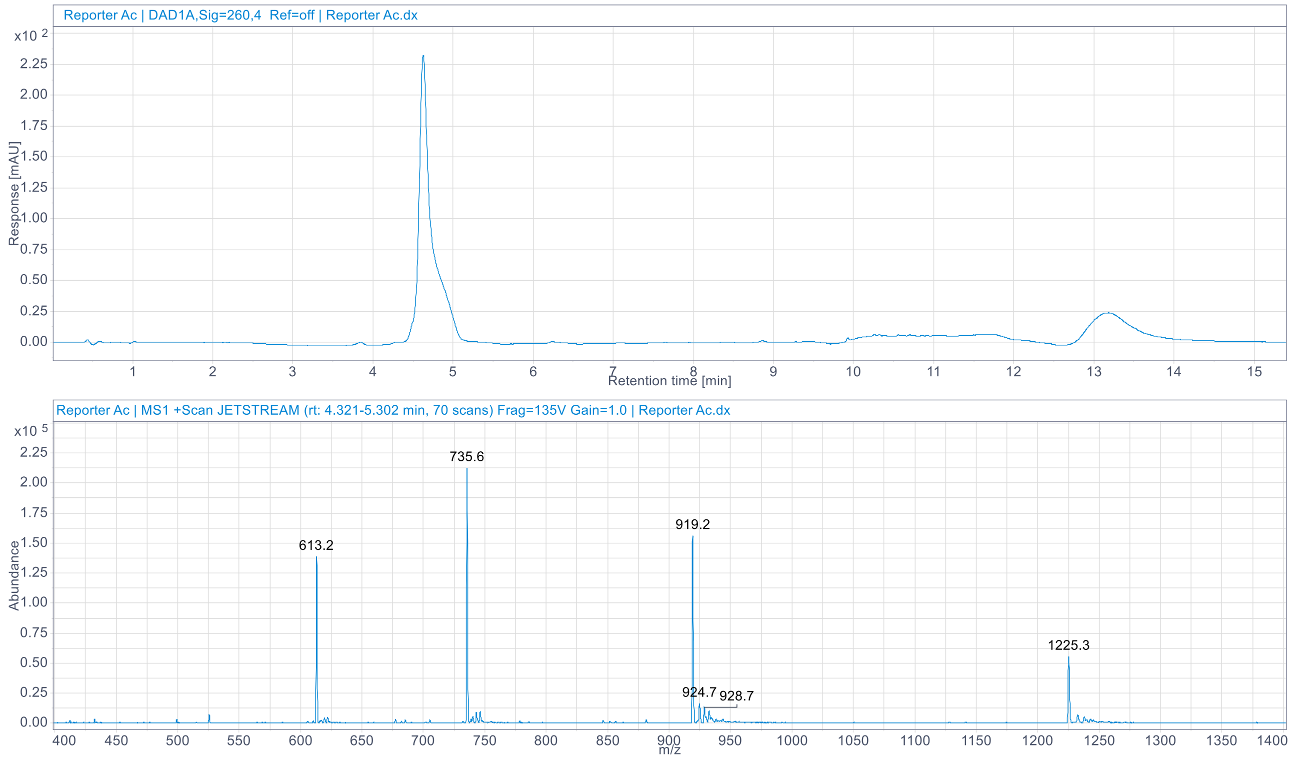

Figure S17. HPLC-UV-MS chromatogram of purified **SA-SP-11-Ac**. HPLC-UV trace at 260 nm (top) and MS spectrum of the corresponding peak at t_r: 4.83 min (bottom). Calcd MW: 3672.71 Da.

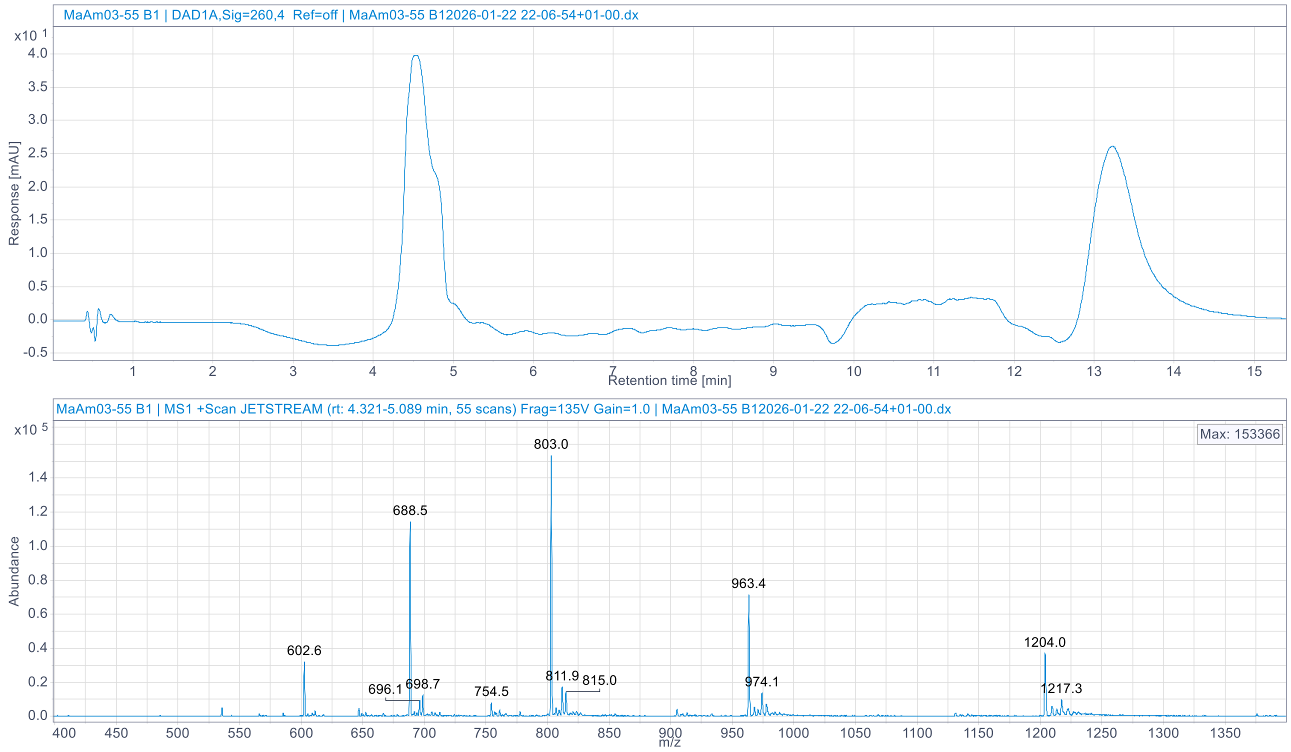

Figure S18. HPLC-UV-MS chromatogram of purified **SA-SP-15-L**. HPLC-UV trace at 260 nm (top) and MS spectrum of the corresponding peak at t_r: 4.54 min (bottom). Calcd MW: 4812.24 Da.

References

1. De La Peña, M. a. G.; Monsalve, R.I.; Batanero, E.; Villalba, M.; Rodríguez, R. Expression in Escherichia Coli of Sin a 1, the Major Allergen from Mustard. *Eur. J. Biochem.* **1996**, *237*, 827–832.
